# Order from Disorder with Intrinsically Disordered Peptide Amphiphiles

**DOI:** 10.1021/jacs.1c06133

**Published:** 2021-07-26

**Authors:** Guy Jacoby, Merav Segal Asher, Tamara Ehm, Inbal Abutbul Ionita, Hila Shinar, Salome Azoulay-Ginsburg, Ido Zemach, Gil Koren, Dganit Danino, Michael M. Kozlov, Roey J. Amir, Roy Beck

**Affiliations:** † Raymond & Beverly Sackler School of Physics & Astronomy, Tel Aviv University, Tel Aviv 6997801, Israel; ‡ The Center for Physics & Chemistry of Living Systems, Tel Aviv University, Tel Aviv 6997801, Israel; ¶ The Center for NanoTechnology & NanoScience, Tel Aviv Univeristy, Tel Aviv 6997801, Israel; § Raymond & Beverly Sackler School of Chemistry, Tel Aviv University, Tel Aviv 6997801, Israel; ∥ Faculty of Physics and Center for NanoScience, Ludwig-Maximilians-Universität, München D-80539, Germany; ⊥ CryoEM Laboratory of Soft Matter, Faculty of Biotechnology and Food Engineering, Technion-Israel Institute of Technology, Haifa 3200003, Israel; # Guangdong-Technion Israel Institute of Technology, Shantou, Guangdong Province 515063, China; @ Sackler School of Medicine, Tel Aviv University, Tel Aviv 6997801, Israel

## Abstract

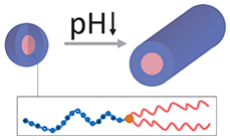

Amphiphilic molecules and their self-assembled structures have long been the target of extensive research due to their potential applications in fields ranging from materials design to biomedical and cosmetic applications. Increasing demands for functional complexity have been met with challenges in biochemical engineering, driving researchers to innovate in the design of new amphiphiles. An emerging class of molecules, namely, peptide amphiphiles, combines key advantages and circumvents some of the disadvantages of conventional phospholipids and block copolymers. Herein, we present new peptide amphiphiles composed of an intrinsically disordered peptide conjugated to two variants of hydrophobic dendritic domains. These molecules, termed intrinsically disordered peptide amphiphiles (IDPA), exhibit a sharp pH-induced micellar phase-transition from low-dispersity spheres to extremely elongated worm-like micelles. We present an experimental characterization of the transition and propose a theoretical model to describe the pH-response. We also present the potential of the shape transition to serve as a mechanism for the design of a cargo hold-and-release application. Such amphiphilic systems demonstrate the power of tailoring the interactions between disordered peptides for various stimuli-responsive biomedical applications.

## Introduction

The self-assembly of amphiphilic molecules holds great interest from a fundamental scientific point of view as well as for their potential for creating nanocarriers for various applications, ranging from drugs and nucleic acids in medicine to fragrances and other small chemicals in the food and cosmetics industries.^1^ The functionality of nanocarriers’ design will depend on many factors, including the mechanism for cargo hold-and-release, biocompatibility, uniformity, and tunability, all of which present challenging obstacles in engineering efficient nanocarriers. Natural lipids and synthetic block copolymers are two of the most widely used amphiphiles in designing such systems, each prevailing due to its specific advantages.^2−12^

An emerging class of synthetic amphiphiles, namely, peptide amphiphiles (PA), is designed to self-assemble into functional structures by building upon the advantageous characteristics of lipids and block copolymers.^13−15^ The molecules’ hydrophilic domain, usually a bioinspired peptide engineered to fulfill one or more active roles, is chemically conjugated to a hydrophobic tail group, usually single or double chain fatty acids like those found in lipids. Due to their highly flexible design scheme, PA self-assemblies can act as organic scaffolds for bone-like mineralization,^16^ anisotropic actuators mimicking skeletal muscle,^17^ produce new versatile soft materials,^18^ and enhance neural progenitor cell differentiation into neurons.^19^ PA can contain spacers or linkers in their design, connecting the hydrophobic tails to the functional hydrophilic domain^20,21^ or conjugating to an ”external” functional group, such as an MRI contrast agent.^22^ Comprehensive research is done to understand the properties and roles of the different molecular domains and their contribution to the specific self-assembled mesophase.^23,24^ In many cases, the bioinspired peptides are designed or derived from proteins with some degree of secondary structure.^25^ Nonetheless, PA hydrophilic domains are not exclusively bioinspired and can be made of any polypeptide sequence, including intrinsically disordered (i.e., unfolded) peptide sequences.

An increasingly large number of proteins have been found to lack a fixed or ordered structure.^26^ As such, these proteins have been termed intrinsically disordered proteins (IDPs). A more operational definition of an IDP is a protein that does not possess only a single functional conformation, but rather, it can fold into an ensemble of functional conformations depending on the setting. This structural plasticity is, in many cases, the result of relatively weaker and transient interactions between proteins segments.^27−30^ The nature of their interactions makes IDPs better suited for specific roles than structured proteins as they can interact with multiple partners or retain in liquid condensed phase.^31,32^

The envisioning of systems with intricate and precise self-organization potential, as sought after in applications, requires the fabrication and processing of unique nanostructures. A primary limitation of current block copolymer synthesis is the lack of general methods for producing precise chain structure (i.e., sequence control) to facilitate multiple desired functions. Natural lipids also have limited functionality due to their relatively small hydrophilic domain. In contrast, intrinsically disordered peptide amphiphiles (IDPAs) benefit from the use of relatively short sequences that are easier to synthesize while still retaining rich functionality. PAs can combine the functionality and flexibility of peptides; since there are 20 natural amino acids, there are practically numerous (>10^9^) possible sequences, even for short (18mers) peptides such as the one studied in this work. Naturally, the design of IDP based hydrophilic domains can be inspired by biology, utilizing the immense pre-existing knowledge base of proteins. To date, only few examples demonstrated exciting functionality using disordered domain in PA.^17,19,20,33,34^

Here, we study the self-assembly and encapsulation capabilities of two IDPAs, composed of hydrophilic domain inspired by the neurofilament-low disordered tail domain and a dendritic branching unit used for conjugating the lipophilic tails.^28,35,36^ Using turbidity, Small-angle X-ray Scattering (SAXS) and cryogenic transmission electron microscopy (cryo-TEM) measurements, we found that the IDPAs self-assembled into well-defined, low-dispersity nanoparticles. In addition, we show that the IDPAs’ sensitivity to pH leads to a tunable and robust organization of the self-assembled nanoparticles. We further show that minor alterations in the peptide sequence can lead to alteration in IDPA–IDPA interaction and the macroscopic arrangement. Last, we demonstrate the potential of using the pH induced shape transition as a release mechanism for the design of nano carriers.

## Results and Discussion

### Synthesis and Structure of IDPAs

**Figure 1:**
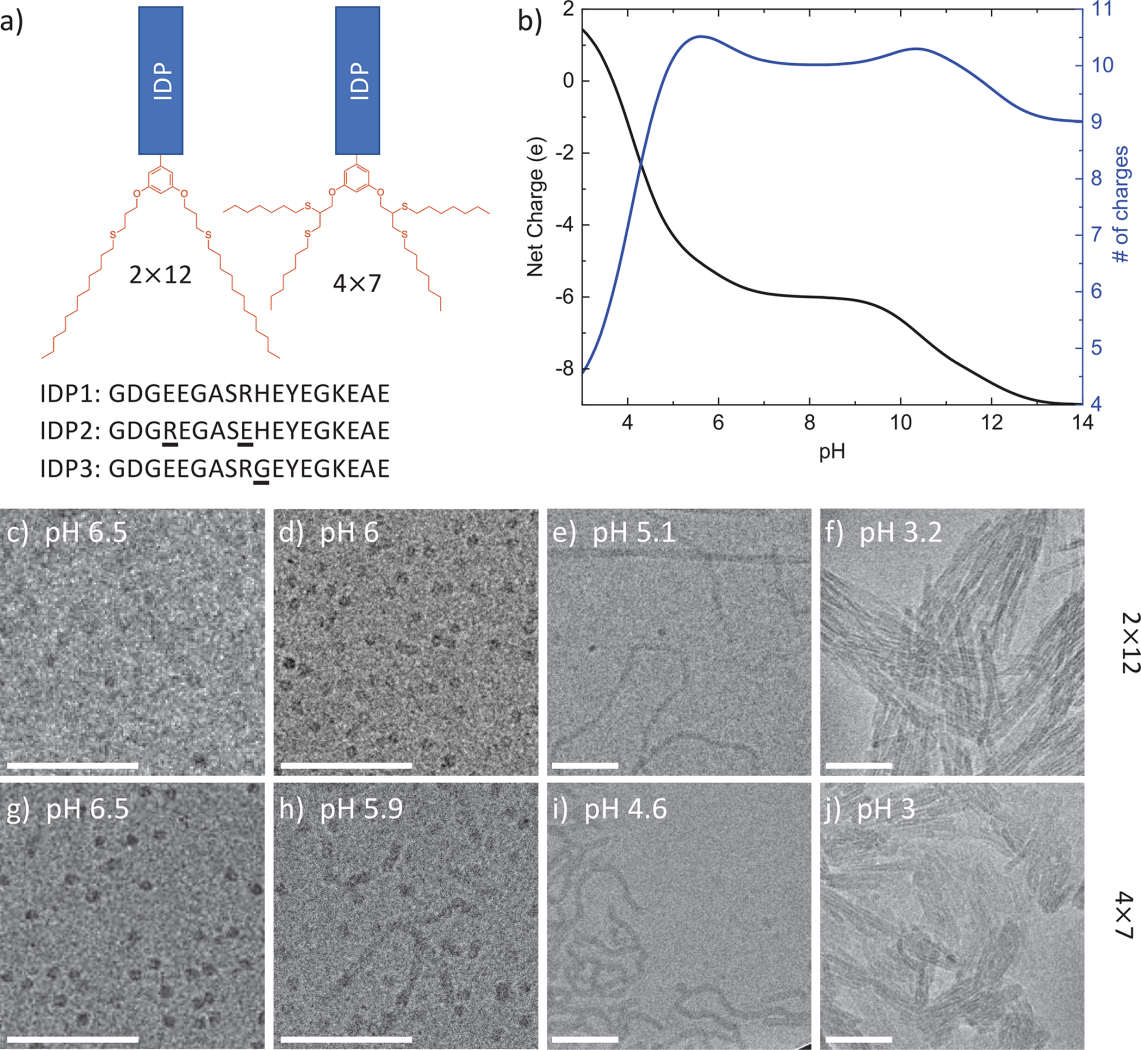
(a) Schematics of the IDPAs with two tail variants (2 × 12 and 4 × 7) and the one-letter IDP’s sequences used in this study. (b) Net charge (black) and the number of charged amino acids (blue) of *IDPA1* hydrophilic domain as a function of pH. (c-j) *IDPA1* Cryo-TEM images showing of self-assembly of (c-f) 2 × 12 and ( g-j) 4 × 7 at various marked pHs. Cryo-TEM images show (c,d,g) spherical micelles at low pH, (e,h,i) coexistence with worm-like micelles at intermediate pH, and (f,j) aggregated micellar rods at low pH. All images were taken at 10 mg/mL IDPAs’ concentration. Scale bar is 100 nm.

IDP sequences were synthesized on an automated solid-phase peptide synthesizer using Fmoc-protected amino acids. The hydrophilic peptide domain sequence is an 18mer amino-acid polyampholyte, inspired by the intrinsically disordered carboxy domain of the protein neurofilament-low (NF-L).^28,29,36,37^ Once the IDP sequences synthesis was completed, an aromatic branching unit containing two allyl or propargyl functionalities and a carboxylic acid was used to cap the N-terminus of the IDP sequence. After the branching units were conjugated, the capped peptides were cleaved from the resin using TFA, and hydrophobic end-groups containing thiols (dodecane-thiol and heptane-thiol) were conjugated to the allyl or propargyl moieties through thiol-ene or -yne click reactions, respectively.^38−41^ We term the tail-group variants by 2 × 12 and 4 × 7 to represent the number and length of the alkyl chains and the amphiphiles as *IDPA1* (Figure 1a). The one letter amino acid sequence of *IDPA1* is GDGEEGASRHEYEGKEAE.

Notably, the peptide’s sequence includes 11 protonatable residues, allowing for the net charge of the peptide to vary significantly as a function of pH (Figure 1b). Specifically, at approximately pH 5.5, there is a decrease in the net charge and in the number of charged residues due to Aspartic Acid and Glutamic Acid residues’ protonation. The critical micelle concentrations (CMC) of 11 μM (=0.03 mg/mL) were determined using the solvatochromic dye Nile Red (Supporting Information Figure S1). A CMC of 5 μM is in the typical micromolar range for peptide amphiphiles.^21,42−44^

The peptides’ degree of disorder was experimentally verified by measuring the circular dichroism (CD) spectrum of samples of the peptides (unconjugated) and the two *IDPA1* variants (Supporting Information Figure S2). In addition, the peptide sequences display a high probability for disorder and the absence of regular secondary structure using the NetSurfP-2.0 bioinformatic algorithm (Supporting Information Figure S3).^45^

### Micellar Nanostructures at High pH

**Figure 2:**
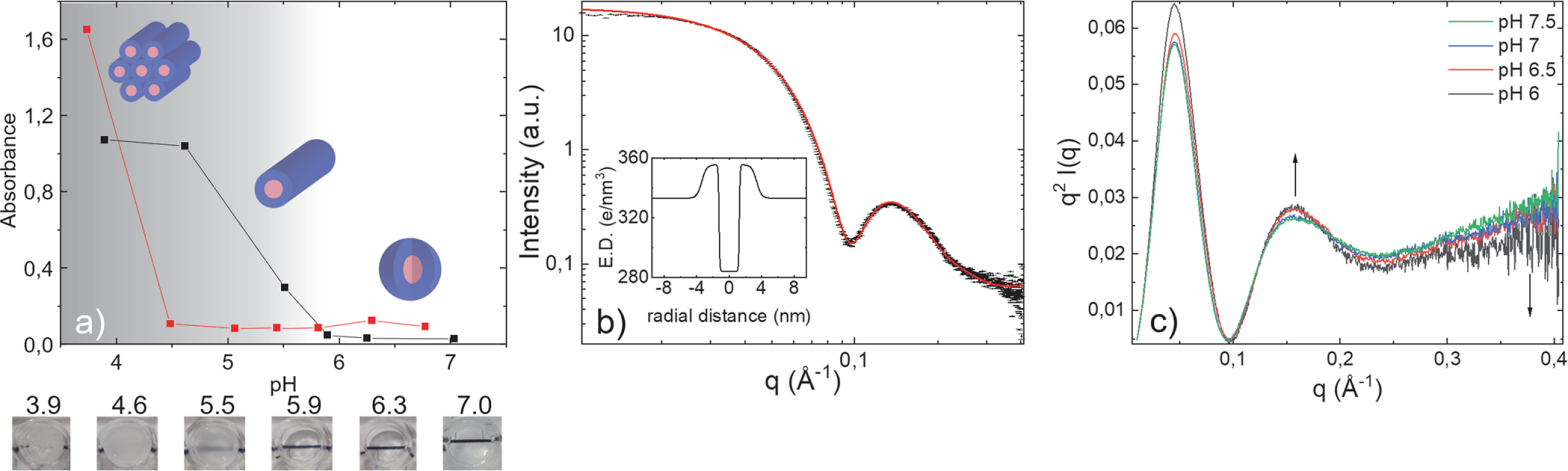
(a) Turbidity measurement of 4 × 7 (black) and 2 × 12 (red) *IDPA1* with schematic representation of phase transition from spherical to cylindrical micelles. Results show an increase in turbidity when lowering the pH, indicating a transition into large assemblies. Turbidity measurements were taken at concentration of 5 mg/mL. Below: photographs of the 4 × 7 *IDPA1* samples measured in the experiment (numbers above photos indicate the pH). b) Spherical core–shell form-factor fit for the SAXS data. Inset, electron density profile used in the fit. c) Kratky analysis with a bell-shaped curve at lower *q*, corresponding to 3D nature of the micelle at larger length-scales, and linear increase at larger *q* resulted from the unfolded state of the peptides at smaller length-scales. SAXS fit is done at 2.5 mg/mL. Kratky analysis is done for 10 mg/mL samples.

We expected *IDPA1* to show little to no sensitivity at high pH where the peptides’ net charge state remains constant (Figure 1b). Indeed, above pH ∼ 6, we find that both tail-variants assemble into nanoscopic spherical micelles, which were visualized via cryo-TEM (Figure 1c-j). At this slightly acidic pH, the micelles showed repulsion and remained miscible at a relatively high IDPA concentration (10 mg/mL). SAXS revealed low-dispersity spherical nanostructures, typical for structured particles. A core–shell form-factor was used to fit the SAXS data and provided the radius of the hydrophobic core *R*_*core*_ = 1.25 ± 0.09 nm, the width of the peptide shell (hydrophilic domain region surrounding the core) *w*_*shell*_ = 2.12 ± 0.05 nm, and the respective average electron densities, ρ_*core*_ = 284 *e*/*nm*^3^ and ρ_*shell*_ = 355 *e*/*nm*^3^ (Figure 2b). Using the SAXS fit and the values from Harpaz et al.,^46^ we estimate the aggregation number for 4 × 7 and 2 × 12 *IDPA1* at pH 7.5 to be approximately 13 and 40, respectively.

The SAXS measurements further revealed the composite dimensionality of the spherical micelles. Using the Kratky analysis,^47^ we found a bell-shaped curve at lower *q*, which corresponds to the 3D nature of the micelle at larger length-scales. However, the linear increase at larger *q* suggests an unfolded state of the peptides at smaller length-scales (Figure 2c). Approaching pH ∼ 6, we find a mild alteration in the SAXS signal, indicating a structural rearrangement at lower pH that we discuss next.

### pH Induced Phase-Transition

The peptide sequence is a strong polyampholyte; hence, the self-assembly of *IDPA1* is expected to depend on pH. Therefore, lowering the pH toward the pI can facilitate a structural phase-transition that depends on the IDP charge density and the amphiphiles’ electrostatic interaction. Turbidity measurements performed on both IDPAs revealed a clear difference in sample translucency above and below pH 6 (Figure 2a). This transition indicates a macro-molecular aggregation of the self-assembled structures at low pH. Below pH 6, the nanostructures interact to produce larger and more ordered aggregates. Figure 2a showcases the transition from translucent to opaque solutions. The turbidity of both variants changes abruptly at pH∼ 6 and 4.5, for the 4 × 7 and 2 × 12, respectively. Notably, the pH-dependent CD measurements (Supporting Information Figure S2) confirm the disorder–order prediction^48^ that these peptides do not undergo any structural reorganization around pH ∼ 6.

Cryo-TEM micrographs of *IDPA1* variants in the pH range 3–6.5 confirm the microscopic transition hinted at by the turbidity measurements (Figure 1c-j). The micrographs show a transition from spherical micelles at pH 6.5 to elongated worm-like micelles lower than pH 5.5. Furthermore, at lower pHs the worm-like micelles are strongly interacting and aggregating. In the case of 4 × 7, the image taken at pH 5.9 nicely shows the coexistence of spherical and worm-like micelles (Figure 1h). In addition, the sphere-to-rod transition and micellar growth is earlier for the 4 × 7 variant and is consistent with its higher hydrophobicity and turbidity measurements.

**Figure 3:**
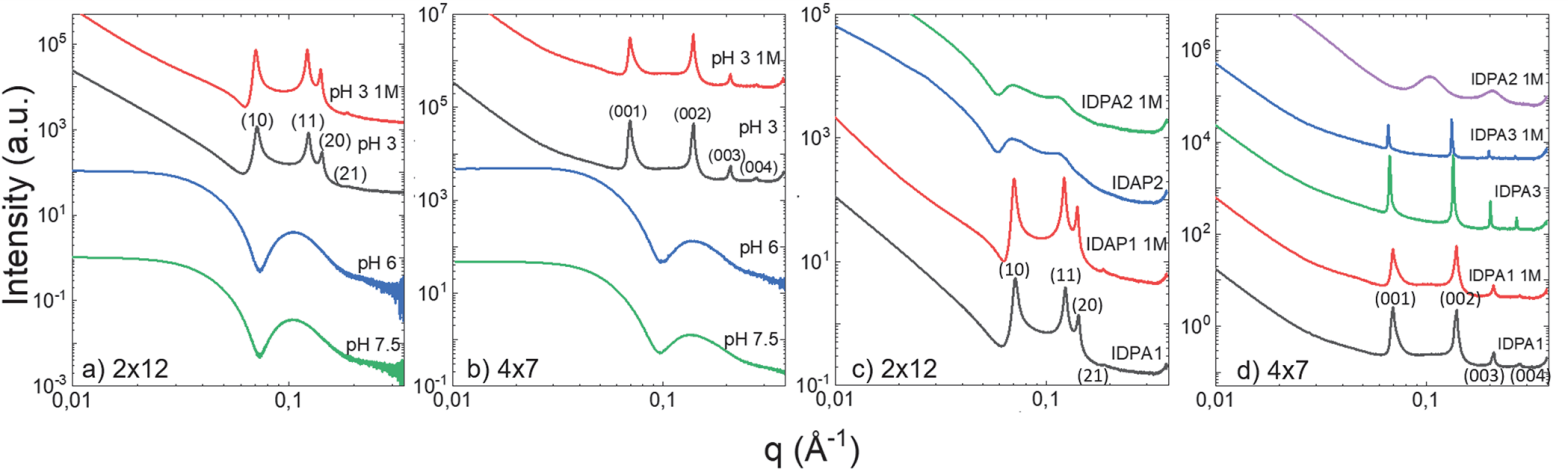
SAXS measurements of (a) 2 × 12 and (b) 4 × 7 *IDPA1* variants at different pHs. Above pH 6, the scattering profile pertains to spherical micelles. Below pH 6, the scattering is dominated by a structure-factor. The 2 × 12 variant forms a hexagonal phase with a spacing of *d*_*H*_ = 10.2 nm, stable at 1 M NaCl (red line). For the 4 × 7 variant, the scattering at low pHs is dominated by a 1D phase structure with a spacing of *d*_*L*_ = 9 nm. Hexagonal and lamellar phase harmonics are indicated by their Millers’ indices in brackets. (c) Comparison of SAXS measurements of 2 × 12 for *IDPA1* and *IDPA2* at pH 3, in either 150 mM salt or 1 M (labeled). The small change in charge distribution has a dramatic effect on the interaction of the worm-like micelles. The sharp structure-factor peaks are replaced with wide and shallow peaks, indicating weaker correlations. (d) Comparison of SAXS measurements of 4 × 7 variants at pH 3, in either 150 mM salt or 1 M (labeled). The mesophase remains the same for *IDPA1* and *IDPA3*, while the *IDPA2* variant shows a pronounced weakening of the intermicelle correlations. All SAXS measurements were taken at 10 mg/mL IDPAs.

Further verification of the phase transition is clearly shown using SAXS (Figure 3a,b). At low pH, the scattering is qualitatively different and is no longer a sum of independent spherical scatterers producing a form-factor SAXS signal. Instead, the SAXS signal now includes an additional structure-factor signal produced by the interparticles’ correlation. The structure-factor peaks position match a 2D hexagonal lattice for 2 × 12 and a 1D lattice for 4 × 7 with the corresponding unit-cell spacing of *d*_*H*_ = 10.2 nm and *d*_*L*_ = 9 nm. The highly dense packing is also evident by the cryo-TEM micrographs showing organization of the micellar rods at low pHs (Figure 1f,j).All structural results are summarized in Table 1.

**Table 1:** Summary of Structural Findings for 2 × 12 and 4 × 7 Variants^a^

				*R*_*sph*_		*R*_*cyl*_		Condensed phase		
Tail	IDPA MW	CMC	pH_*T*_	SAXS	TEM	SAXS	TEM	Symmetry	Unit cell, SAXS	*D*_*NN*_
	[kDa]	[μM]		[nm]	[nm]	[nm]	[nm]		[nm]	[nm]
2 × 12	2.57	5	5.9	4.7	4.4	4.1	4.2	Hexagonal	10.2	10.6
4 × 7	2.69	11	4.5	3.4	3.9	4.2	3.8	Lamellar	9	10

a *pH*_*T*_ is the turbidity onset, *R*_*sph*_ and *R*_*cyl*_ denote spherical and cylindrical radii, respectively, as measured by SAXS and cryoTEM (fits for SAXS in Supporting Information Figure S12). For the condensed phase, the observed symmetry was measured by SAXS with the relevant unit cell spacing. *D*_*NN*_ denotes nearest neighbour spacing measured by cryoTEM.

### Engineering Self-Assembly by Point Mutation

The added value of using peptides as the hydrophilic domain is the possibility to tune the interactions via small alterations in the sequence, such as a single mutation. We recently demonstrated that a similar sequence peptide alters its self-interaction via a single point mutation.^49^ Short-ranged transient interactions are also present in the original neurofilament disordered protein,^28,29,50−53^ presumably due to the oppositely charged amino-acids along the polyampholytic intrinsically disordered C-terminus domain. We synthesized a sequence variant by changing the Glutamic Acid at position 4 with the Arginine at position 9, termed *IDPA2*. By doing so, we are conserving the net charge and the functional relation between pH and charge but altering the charge distribution at the hydrophilic peptide domain. Such alteration is expected to alter the ionic bridging between the IDPs.^28,50,53^

In addition, when considering the observed structural pH sensitivity, with a phase-transition being located somewhere between pH 4 and 6 (Figures 1c,d and 3), a possible origin for the transition can be the charging state of the Histidine amino-acid. To verify this hypothesis, we designed a slightly modified hydrophilic domain sequence, termed *IDPA3*, that replaces the Histidine at position 10 with a neutral and pH insensitive Glycine residue. Notably, the two new IDPA variants are still considered highly disordered and are assigned the random coil conformation by the predictors (Supporting Information Figure S4).

Indeed, we found that the hydrophilic domain (i.e., the disordered peptide) and its interactions in the assembly control the complex aggregations at low pH. SAXS experiments show that replacing the Histidine with a Glycine served to strengthen the interaction between the worm-like micelles (Figure 3d). However, slightly changing the sequence’s order, namely, the *IDPA2* variant, noticeably changes the complex aggregation (Figure 3c). For the 2 × 12 variant, we saw a significant weakening of micelle–micelle interactions in the worm-like phase, at 150 mM and 1 M salt concentration, demonstrated by the diminished structure-factor scattering (Figure 3a). However, the 4 × 7 variant showed a similar weakening of the interactions only at 1 M salt pointing toward nontrivial electrostatic interaction^27,28,53^ between the IDPs (Figure 3b).

### Cargo Encapsulation and Release

**Figure 4:**
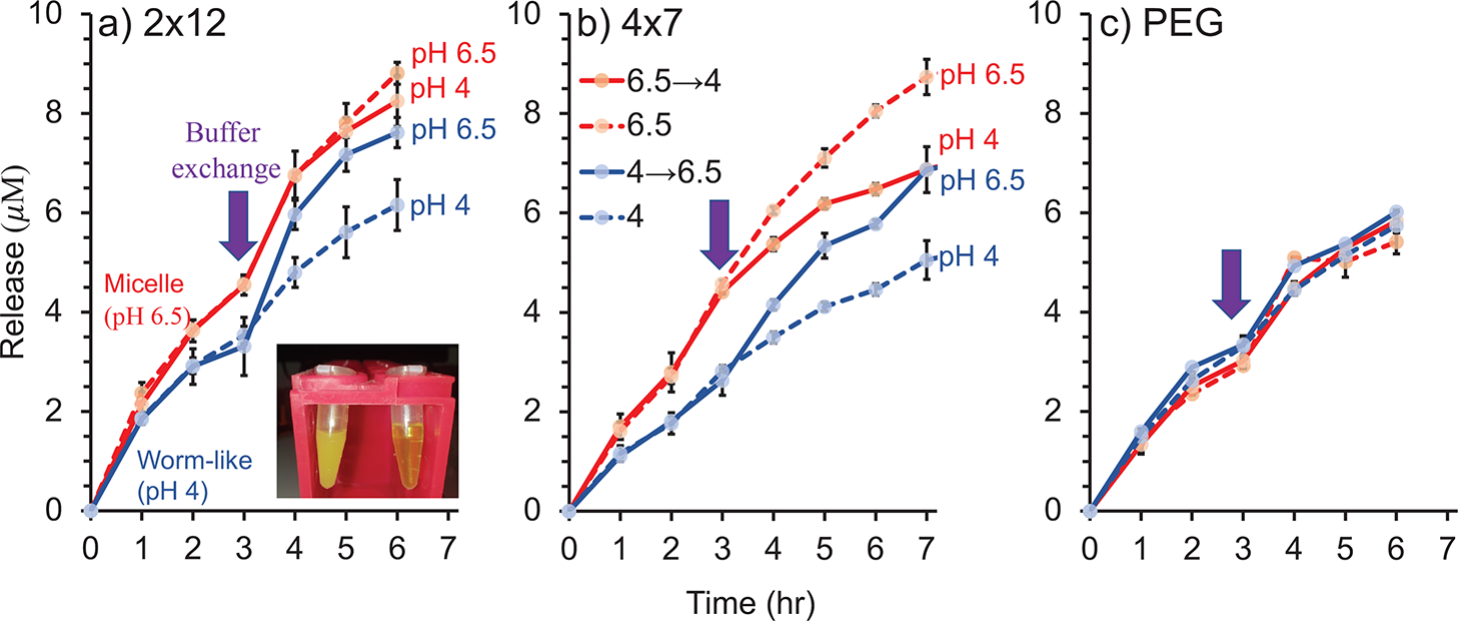
Encapsulation measurements. Accumulative released dye concentration for (a) 2 × 12, (b) 4 × 7 IDPAs and (c) PEG-2 × 12. Blue and red data-points represent a baseline pH for the experiments of 6.5 and 4, respectively. After 3 h, the buffer was exchanged either to induce structural mesophase transition via pH trigger (solid lines), or to identical and fresh buffer (dashed line). PEG-2 × 12 amphiphiles at pH 6.5 and pH 4 show no pH release trigger. Inset, representative photo of the encapsulated dye in (left) worm-like micelles at pH 4 and (right) spherical micelles at pH 6.5. Encapsulation experiments were done with 5 mg/mL IDPA in buffer at pH 6.5 with 2 mM of DMSO dye.

After the pH-depended self-assembly into micelles and worm-like micelles was characterized, we wanted to evaluate how the shape transition will affect encapsulated cargo release. We chose butyl ester of 7-(diethylamino)coumarin-3-carboxylic acid as the hydrophobic cargo and used a dialysis setup to study the hydrophobic dye’s release from the assembled structures. Solutions containing micelles of 2 × 12 and 4 × 7 IDPAs (pH 6.5) were mixed with a stock solution of the dye, followed by filtration of the residual unencapsulated dyes. Next, the samples’ pH was adjusted to pH 4 by adding a few μL of HCl to transform the spherical micellar assemblies into the worm-like micelles. As expected, while the micelle solution was clear, the worm-like micelle solution became highly turbid (Figure 4).

Next, the two solutions were transferred into dialysis tubes and placed in buffer solutions (pH 6.5 or 4 for the micelles and worm-like micelles, respectively) containing bovine serum albumin (BSA). The BSA’s role was to scavenge the released dyes and avoid their aggregation and dissolution due to their poor aqueous solubility. The solutions were then placed in a shaking incubator at 37 °C, and samples were taken periodically from the outer solution and characterized by a spectrophotometer to determine the released dye’s concentration.

We find that the spherical micelles’ release was faster than the release from the worm-like assemblies (Figure 4). To quantify how the phase-transition affects the release, we continued measuring the release after the solutions’ pH was adjusted from 6.5 to 4 or vice versa, and the samples were placed in suitable fresh buffers. As controls, we used samples kept under the same initial pH and placed them into fresh buffers. It was fascinating to see the change in release rate due to the pH-induced shape transition as worm-like micelles that were transformed into spherical micelles (solid blue lines in Figure 4a,b) started to release the dyes faster, showing a similar rate as the nonaltered micelle control (dashed red lines in Figure 4a,b). Simultaneously, a slower release was observed for spherical micelles, which were transformed into worm-like micelles (solid red lines and dashed blue lines in Figure 4a,b). The changes in release rate due to the pH-induced change in mesophases were observed for both 2 × 12 and 4 × 7 IDPAs, with the effect being more significant for the latter. To ensure that the release rates were genuinely affected by the change in the assemblies’ shape, we prepared a nonresponsive amphiphile with two dodecane alkyl chains by replacing the hydrophilic IDP with a PEG chain of similar molecular weight. The PEG-based amphiphile (PEG-2 × 12) self-assembled into micelles with a diameter of hydration of around 10 nm at both high and low pH (Supporting Information Figure S5), demonstrating that its assembly is not responsive to pH at the tested range. We were encouraged to see that the control amphiphile release rates were not affected by the pH-jump after 3 h (Figure 4c). These results indicate that it is indeed the change in the shape of the IDPA assemblies due to altered interaction between the hydrophilic peptide domains, which affects the release rate.

### Discussion

We demonstrated the IDPA could serve as a valuable platform having stimuli-responsive self-assembly. Importantly, these nanoscopic assemblies are well-ordered, although originating from disordered peptides. When modeling the IDPA interactions and phase-transitions presented here, it is essential to understand whether the dominant interactions are nonspecific, e.g., based on the total charge-density, or specific, e.g., conformational-based interactions. A generic model for a spherical-to-cylindrical micelle phase-transition will be agnostic to the charged peptide domain’s details, aside from the charge distribution.^54^ Such a model, detailed for brevity in the Supporting Information, describes the free-energy balance between the energy associated with the hydrophilic region’s charge-density and the energetic price paid for inducing a curvature that deviates from the intrinsic one. Essentially, lowering the pH results in a lower net charge that allows the hydrophilic peptide to compress. Assuming that the intrinsic curvature favors cylindrical morphology, this can facilitate the decrease in bending energy with a denser hydrophilic region.

Our generic theoretical arguments can explain, to a first-order, the difference in pH response of the two hydrophobic tails varients. Specifically, to convert worm-like to spherical micelles, stronger electrostatic repulsion between the hydrophilic chains is needed. It is clear that the differences between the 2 × 12 and 4 × 7 pH turbidity response and mesophase transitions are related to the “effective bulkiness” of the hydrophobic domain. In particular, compared to the 2 × 12 variants, the 4 × 7 contains additional hydrocarbons, and the chains are condensed closer to the aromatic ring. Such architecture increases the tendency to form cylinders over spherical micelles from packing reasons.^55^ As shown in Figure 2a, the transition from spherical micelles to cylinders, and condensation of the latter, occur at higher pHs (i.e., a higher net charge) for the 4 × 7 than for the 2 × 12 variants.

However, the mutated variants’ experimental finding suggests that we must also consider the sequence’s details and charges distribution along the peptide chain. Therefore, a sequence-dependent interaction term must be included in the free-energy calculation, describing the interaction between the amino-acids along two parallel and offset peptides^28,36,53^ (Supporting Information).

The experimental results on the *IDPA2* variant show that indeed such a small change significantly affects the intermicelle interactions, and as a result, the aggregation phase was dramatically changed. Therefore, the minute alteration between the original sequence and its variants suggests sequence-specific interactions and most likely interdigitation between neighboring particles. Moreover, the entire IDPA architecture, including the hydrophobic domain, also plays a role in the pH-response and the macroscopic aggregated mesophase (i.e., at low pH). For the 4 × 7 hydrophobic domain, there is a clear structural difference between the *IDPA2* and the other variants at high salt concentration. For the 2 × 12 hydrophobic domain, while the condensed phase structural correlation weakens for the *IDPA2* variants, they are salt-independent between 0.15 and 1 M. Indeed, as suggested by our theoretical modeling, the *IDPA3* condensed phase (at pH = 3) is somewhat similar to the original variant (see Supporting Information).

From our X-ray data, we find that the core radii are 1.25 and 2.0 nm for the 4 × 7 and 2 × 12 variants, respectively. The maximum extension for *n* hydrocarbons follows *l*_*max*_ = (0.154 + 0.1265*n*) × *nm*.^55^ Addition of the length of the benzene ring makes the maximum core radii 1.61 and 2.42 nm for the 4 × 7 and 2 × 12 variants, respectively. Therefore, the core is smaller than the maximum extension of each chain, but not by much. Regardless, our data cannot distinguish between hydrocarbon chains that are interlocked or folded back on themselves.

For the practical point-of-view, the IDPA molecular architecture proved to be very useful as a nanocarrier with stimuli response. Unlike PEG-based particles, upon pH alteration and structural transformation, cargo release is expedited. However, the different structural configurations also differ in their leakiness (i.e., release rate at a fixed condition). Here, spherical micelles show a higher release rate than worm-like micelles. Our structural study found minor differences between the radii of the spherical micelles and the worm-like-micelles. However, the rate of the release must be proportional to the cumulative surface area of the particles. Here, the cumulative surface area is larger for the spherical micelles than for a worm-like micelle containing the same amount of dye. Importantly, this argument is grounded on the fact that the cross-section of both types of micelles is about the same. Furthermore, the stronger repulsion between the peptide chains in the spherical micellar phase may introduce additional water channels allowing the dye to leak out faster than the worm-like-micelles peptide–peptide interaction causes tighter packing.

## Conclusions

We investigated the conjugation of disordered polypeptide domains with hydrocarbon dendrimers into IDPA. We find that the interactions between the peptides lead to tunable self-assembled nanostructures. The IDPA hydrophilic domains are weakly interacting and disordered by nature, generally associated with transient behavior. Nonetheless, the IDPA self-assembly is remarkably forming ordered nanoparticles.

Moreover, the IDPA system shows pH response and structural phase-transition. It can be useful as a trigger for cargo release, similar to already demonstrated amphiphilic polymer-based nanoparticle systems with drug release enzymatic response.^56^

We further demonstrated that minute alteration in the disordered peptide sequence and dendrimer architecture could significantly impact the nanoscopic and macroscopic length-scales. This platform also enables in situ modifications of the IDPs and the study of the mutual interaction between IDPs. Last, as we showed here, modification of the IDP domain, and its interaction with the surrounding, plays a critical role in the IDPA’s self-assembly and structural transformation from external cues. We expect that this platform can be furthered explored for targeted drug-delivery where tailored biological signals will induce phase-transition and expedite release.

## Experimental Section

### Synthesis and Purification

All peptides were synthesized at the Blavatnik Center for Drug Discovery (BCDD) at Tel Aviv University using automated Fmoc solid-phase peptide synthesis using The Liberty Blue automated microwave peptide synthesizer (CEM, Matthews, NC, USA). After the coupling of the last amino acid, either 3,5-bis(allyloxy)benzoic acid or 3,5-bis(prop-2-yn-1-yloxy)benzoic acid^57^ were coupled to the N-termius of the peptide. The capped peptides were cleaved from the resin using standard conditions (95% trifluoroacetic acid (TFA) (v/v), 2.5% H2O (v/v), and 2.5% triisopropylsilane (v/v) for 3 h). The cleaved diallyl or dipropargyl were purified by Waters AutoPurification system (MS directed LC) and were further reacted in thiol–ene or thiol–yne reactions with 1-dodecanethiol or 1-heptanethiol, respectively, as described below to yield IDPAs 2 × 12 and 4 × 7, respectively.

#### IDPA1-Diallyl

Exact Mass 2164.88. Detected Mass: ES mode + : 723.28 [(M+3H)/3] and 1084.21 [(M+2H)/2].

#### IDPA1-2 × 12

A 54 mg (0.025 mmol) portion of peptide 2 was dissolved with 350 μL of phosphate buffer (pH 7.4, 100 mM) using gentle heating, then 1 mL of DMF was added. A 5.1 mg portion of DMPA (0.02 mmol) was separately solubilized with 100 μL of DMF, and the solution was added to the peptide solution followed by the addition of 239.5 μL 1-dodecanethiol (0.997 mmol). The solution was purged with N_2_ for 15 min and then stirred under UV light for 2 h. Next, the crude mixture was placed inside a dialysis membrane with MWCO of 3000 Da and dialyzed against DI water for 12 h. The solution was lyophilized, and final purification was performed using preparative-scale reversed-phase HPLC (Waters AutoPurification system, spectra for HPLC in Supporting Information Figure S7). The product was confirmed by LC/MS. ACN was removed by rotary evaporation, and the solution was further lyophilized yielding a white solid product (31.26 mg) (48% yield). Exact mass 2569.23. Detected mass: ES mode + : 858.17 [(M+3H)/3] and 1286.78 [(M+2H)/2].

#### IDPA1-Dipropargyl

Exact Mass 2160.85. Detected mass: ES mode + : 721.91 [(M+3H)/3] and 1082.23 [(M+2H)/2].

#### IDPA1-4 × 7

A 58 mg (0.027 mmol) portion of peptide 1 were dissolved with 350 μL of phosphate buffer (pH 7.4, 100 mM) using gentle heating, followed by the addition of 1 mL of DMF. A 5.48 mg portion of 2,2-dimethoxy-2-phenylacetophenone (DMPA, 0.021 mmol) was separately solubilized with 100 μL of DMF and added to the peptide solution flowed by addition of 338 μL 1-heptanethiol (2.1 mmol). The solution was purged with N_2_ for 15 min and then stirred under UV light for 2 h. Next, the crude mixture was placed in dialysis membrane with MWCO of 3000 Da and dialyzed against DI water for 12 h. The solution was lyophilized, and the product was purified using preparative-scale reversed-phase HPLC (Waters AutoPurification system, spectra for HPLC in Supporting Information Figure S8). The product was confirmed by LC/MS. ACN was removed by rotary evaporation and further lyophilized yielding a white solid product (19.2 mg) (26% yield). Exact Mass 2689.24. Detected Mass: ES mode + : 898.22 [(M+3H)/3] and 1346.78 [(M+2H)/2].

#### IDPA2-Diallyl

Exact Mass 2164.88. Detected Mass: ES mode + : 723.26 [(M+3H)/3] and 1084.19 [(M+2H)/2].

#### IDPA2–2 × 12

Was synthesized similarly to IDPA1. A 51 mg (0.024 mmol) portion of IDP2- were mixed with 4.8 mg of DMPA and 226 μL of 1-dodecanethiol and reacted and purified as was described for the synthesis of IDPA1 to yield 30.7 mg (50% yield). MS analysis: Exact Mass: 2569.23 Detected Mass: ES mode + : 858.17 [(M+3H)/3] and 1286.78 [(M+2H)/2].

#### IDPA2-Dipropargyl

Exact Mass 2160.85. Detected Mass: ES mode + : 721.89 [(M+3H)/3] and 1082.14 [(M+2H)/2].

#### IDPA2–4 × 7

Was synthesized and purified similarly to IDPA1. A 54 mg (0.025 mmol) portion of IDP2-dipropargyl were mixed with 5.1 mg of DMPA and 315 μL of 1-heptanethiol and reacted and purified as was described for the synthesis of IDPA1 to yield 26.8 mg (40% yield). MS analysis: Exact Mass: 2689.24 Detected Mass: ES mode + : 1346.78 [(M+2H)/2] and 898.22 [(M+3H)/3].

#### IDPA3-Dipropargyl

Exact Mass 2080.81. Detected Mass: ES mode + : 695.06 [(M+3H)/3] and 1041.95 [(M+2H)/2].

#### IDPA3-4 × 7

Was synthesized and purified similarly to IDPA1. Fourteen mg (0.0067 mmol) of IDP3-dipropargyl was mixed with 1.36 mg of DMPA and 84 μL of 1-heptanethiol, and the mixture was reacted and purified as was described for the synthesis of IDPA1 to yield 9 mg (51%). MS analysis: Exact Mass: 2609.20 Detected Mass: ES mode + : 1306.83 [(M+2H)/2] and 871.47 [(M+3H)/3].

#### PEG-Dendron (2 × 12) Synthesis

Dendron: 600 mg (2.5 mmol) of 3,5-(diallyloxy) benzoic acid,^57^ 3.11 g of 1-dodecanethiol (15 mmol), and 38.4 mg of 2,2-dimethoxy-2- phenylacetophenone (DMPA; 0.15 mmol) were dissolved in 800 μL of DMF. The solution was purged with N_2_ for 15 min and then stirred under UV light for 2 h. Next, the crude mixture was loaded on a silica column; thiol excess was washed with 10:90 ethyl acetate and hexane (v/v), and 2 × 12 dendron compound was eluted with 30:70 ethyl acetate and hexane (v/v). The fractions that contained the product were unified, evaporated, and dried under high vacuum obtaining 1.5 g of yellowish oily compound (92% yield). ^1^H NMR (400 MHz, chloroform-*d*) δ 7.23 (d, *J* = 2.2 Hz, 2H), 6.69 (t, *J* = 2.3 Hz, 1H), 4.10 (t, *J* = 6.1 Hz, 4H), 2.70 (t, *J* = 7.1 Hz, 4H), 2.52 (t, *J* = 7.0 Hz, 4H), 2.07 (p, *J* = 6.5 Hz, 4H), 1.59 (p, *J* = 7.3 Hz, 4H), 1.26–1.38 (m, 37H), 0.88 (t, *J* = 6.8 Hz, 6H). PEG-dendron (2 × 12): 70 mg of 2 kDa PEG-amine^58^ were dissolved in 100 μL of DCM and 67.3 mg of 2 × 12 dendron, and 40 mg of (2-(1H-benzotriazol-1-yl)-1,1,3,3-tetramethyluronium hexafluorophosphate HBTU was dissolved in DCM:DMF 1:1 (1 mL) followed by the addition of DIPEA; 60 μL was added to the PEG-amine solution, and the mixture was allowed to stir for 3 h at room temperature. The crude mixture was loaded on a MeOH based LH20 SEC column. The fractions that contained the product were unified, and the MeOH was evaporated in vacuum to obtain 76 mg (82%) of the PEG based amphiphile. ^1^H NMR (400 MHz, chloroform-*d*) δ 6.91 (d, *J* = 2.2 Hz, 2H), 6.69 (t, *J* = 5.8 Hz, 1H), 6.57 (t, *J* = 2.2 Hz, 1H), 4.08 (t, *J* = 6.1 Hz, 4H), 3.83–3.45 (PEG backbone), 3.38 (s, 3H), 2.77 (t, *J* = 6.5 Hz, 2H), 2.70–2.63 (m, 6H), 2.52 (t, *J* = 7.4 Hz, 4H), 2.05 (p, *J* = 6.6 Hz, H), 1.88 (p, *J* = 6.6 Hz, 2H), 1.62–1.52 (m, 4H), 1.45–1.26 (m, 40H), 0.88 (t, *J* = 6.7 Hz, 6H).

### SAXS and Cryo-TEM Sample Preparation

The IDPA or peptide powder was first fluidized in purified water (Milli-Q) at twice the desired concentration. The solution was then titrated with NaOH to a pH where the solution became more homogeneous (preferably a pH where the IDPAs are soluble in water). Titration was monitored using a pH probe (Sentek P13 pH Electrode). Following titration, 50 μL of the solution was combined with 50 μL of 2X the buffer of choice to achieve a pH in the vicinity to the desired one. The 2X buffer acetic Acid (pH 3–4.5), MES pH (5–6.5), and MOPS (pH 7–7.5) were prepared at 200 mM to achieve final buffer molarity of 100 mM after being mixed with IDPA or a peptide solution 1:1 (vol/vol). Samples were prepared by diluting the 2X titrated samples with a buffer. This buffer was premade with the different salts. Moreover, samples were equilibrated for a day and then measured. Many days (2–3 weeks) later, additional measurements of the sample after 2–3 weeks from preparation did not show structural rearrangement or an aging effect.

### SAXS

For solubilizing conditions (above the transition pH, generally above pH 6), samples were measured at three synchrotron facilities: Beamline B21, Diamond Light Source, UK, beamline 12.3.1, SIBYLS, Advanced Light Source, Berkeley, USA and beamline SWING, SOLEIL synchrotron facility, Paris, France.

For phase-separating samples that display sediment (below the transition pH, generally pH 3–5.5), measurements were performed using an in-house X-ray scattering system, with a Genix3D (Xenocs) low divergence Cu Kα radiation source (wavelength of λ = 1.54 Å) with a Pilatus 300 K (Dectris) detector and scatterless slits setup^59^ as well as beamline I22 at Diamond Light Source. Here, samples were measured inside 1.5 mm quartz capillaries (Hilgenberg).

#### Peptide’s SAXS Analysis

The unconjugated peptide by itself was measured using SAXS at different pH levels to test the effect on its ensemble-averaged structure. The peptide at each pH was measured at four different concentrations to extrapolate to the noninteracting ”zero-concentration” peptide scatterings. From the low momentum transfer (*q*) regime of the extrapolated zero-concentration scattering curves, we extracted the radius of gyration (*R*_*g*_) and the forward scattering, *I*(0), using the Guinier analysis (Supporting Information Figure S6). When examining the values of *R*_*g*_ from high to low pH, they seemed to remain constant until pH ∼ 5.5 and gradually increase below it. However, this increase in size can be explained by a simultaneous increase in the effective mass of the peptides (increase in forwarding scattering) due to a decrease in interpeptide repulsion near the isoelectric point (pI).

### Cryo-TEM

Cryo-TEM specimen preparation was performed by applying a 6 μL drop of the studied solution to a perforated carbon film supported on a 200-mesh TEM copper grid, thinning (blotting), and removing of excess solution. The procedure was carried out at a controlled temperature (25 °C) and water saturation. Next, the sample was vitrified in liquid ethane at its freezing point (−183 °C). The procedure was carried out at a controlled temperature (25 °C) and water saturation. The vitrified specimens were stored under liquid nitrogen (−196 °C) until examination. The procedure was carried out at a controlled temperature (25 °C) and water saturation. Next, the sample was vitrified in liquid ethane at its freezing point (−183 °C) and transferred to liquid nitrogen (−196 °C) for storage until examination. The samples were then examined using a Tecnai T12 G2 (FEI, The Netherlands) TEM operated at an accelerating voltage of 120 kV, keeping the specimen temperature below −170 °C during transfer and observation. Images were digitally recorded on a Gatan Ultrascan 1000 cooled CCD camera using the Gatan Digital Micrograph software package. Images were recorded using methodologies we developed^60^ under low-dose conditions to minimize electron beam radiation damage. The diameters of the assembly micelles were measured with a digital micrograph and ImageJ. To calculate the repeating distance between rods in the condensed phase, the length of the arbitrary domains containing 5–15 rods were measured; the number of rods in each domain was multiplied by the each rod’s diameter, and the result was subtracted from the length of the domain and divided by the number of rods in the domain.

### Turbidity

All measurements were recorded on a TECAN Infinite M200Pro device. The amphiphiles were treated and prepared in the same manner as previously described to achieve a final concentration of 5 mg mL^–1^. A 100 μL portion of each solution was loaded onto a 96 well plate. The absorbance at 600 nm was scanned for each well.

### CD

Circular dichroism (CD) measurements were performed using a commercial CD spectrometer (Applied Photophysics Chirascan). Both IDPAs, 2 × 12 and 4 × 7 and the unconjugated IDP, were placed in a glass cuvette with a 1 mm path length. The IDPAs and peptide were mixed with a phosphate buffer to achieve a concentration of 0.05 and 0.1 mg mL^–1^, respectively. Measurements were performed using phosphate buffer since the buffers used for the X-ray scattering experiments (mainly MOPS and MES) have high absorbances in the relevant CD wavelengths. The 190–260 nm wavelength range was probed in 1 nm steps, with 0.5 s at each point. Three measurements were performed for each and averaged.

### CMC

The amphiphile was dissolved in the diluent (15 mL of MOPS buffer solution (pH 7.4) and 7.5 μL of Nile red stock solution (2.5 mM in ethanol) were mixed to give a diluent with a final concentration of 1.25 μM) to give a final concentration of 400 μM IDPA, and the mixture was sonicated for 5 min. This solution was repeatedly diluted by a factor of 1.5 with diluent. A 100 μL portion of each solution was loaded onto a 96 well plate. The fluorescence emission intensity was scanned for each well (550 nm emission intensity scan: 580–800 nm) using TECAN Infinite M200Pro plate reader. Maximum emission intensity was plotted vs concentration in order to determine the CMC. This procedure was repeated thrice.

### Cargo release experiment

The encapsulation of the hydrophobic fluorescent dye butyl 7-(Diethylamino)coumarin-3-carboxylate was performed by mixing a micellar solution of IDPA (5 mg/mL; 1.9 mM) in buffer at pH 6.5 with stock solution of the dye (50 mM in DMSO) to get to a final concentration of 2 mM of the dye. To remove any undissolved dye, the solution was filtered using a 0.45 μm Nylon filter, and the clear solutions were analyzed using TECAN Infinite M200Pro plate reader to measure their fluorescence emission (λ_*ex*_ = 400 nm; λ_*em*_ = 435–650 nm). The integrated fluorescence emission intensity values were then compared to a calibration curve. Release experiments of hydrophobic dye (butyl-coumarin) were performed using a dialysis tube (Mini GeBA ex-Kit, 8 kDa MWCO, volume range 10–250 μL. A volume of 80 μL of the micelles and worm-like micelle solutions were placed in the dialysis tubes, and each tube was immersed into 8 mL of buffer (with 0.5 mg/mL of BSA) at pH 6.5 or 4, respectively. The solutions were placed in a shaker incubator at 37 °C and every hour, 100 μL were taken from the outer buffer and placed in a 96 well plate to measure the concentrations of butyl-coumarin dyes outside the dialysis-tube with a TECAN Infinite M200Pro plate reader (λ_*ex*_ = 420 nm; λ_*em*_= 435–650 nm). After 3 hours, the micelle solution (inside the tube) at pH 6.5 was adjusted to pH 4 using HCl (5M) and then the tube was immersed into fresh 8 mL of buffer at pH 4 with 0.5 mg/mL of BSA. A similar process was done to the worm-like solution. In order to transform them to micellar phase, the inner pH 4 solution was adjusted to pH 6.5 by addition of NaOH (5M). Then the tube was immersed into fresh 8 mL of buffer at pH 6.5 with 0.5 mg/mL of BSA. The solutions were placed back in the shaker incubator at 37 °C and every hour, 100 μL were taken from the outer buffer and placed in a 96 well plate to measure the concentrations of butyl-coumarin dyes outside the dialysis-tube by the TECAN Infinite M200Pro plate reader (λ_*ex*_ = 400 nm; λ_*em*_ = 435–650 nm). pH adjustments: the titration was monitored using a pH probe small enough to fit into an Eppendorf (Sentek P13 pH Electrode).

## Supporting Information Available

The Supporting Information includes Figures S1–S12 referred in the main text, chemical structures, HPLC chromatograms and MS data for the different IDPAs (Figures S7–S11), and theoretical arguments for the observed spherical-to-cylindrical micelle phase-transition seen in our system, including Figures S13–S17 referred within the supporting text. The Supporting Information is available free of charge at https://pubs.acs.org/doi/10.1021/jacs.1c06133.
