# Supplementary material for: Order from Disorder with Intrinsically Disordered Peptide Amphiphiles
Source: J Am Chem Soc. 2021 Jul 26;143(30):11879–88. doi: 10.1021/jacs.1c06133 (PMC8397319; doi:10.1021/jacs.1c06133)
Supplement: Supplementary file 1 — ja1c06133_si_001.pdf [file ja1c06133_si_001.pdf]

Order from disorder with intrinsically disordered peptide amphiphiles

Guy Jacoby, Merav Segal Asher, Tamara Ehm, Inbal Abutbul Ionita, Hila Shinar, Salome Azoulay-Ginsburg, Ido Zemach, Gil Koren, Dganit Danino, Michael M. Kozlov, Roey J. Amir, Roy Beck

This file contains:

- Figures S1-S12 referred in the main text
- Chemical structures, HPLC chromatograms and MS data for the different IDPAs (Figurex S7-S11)
- Theoretical arguments for the observed spherical-to-cylindrical micelle phase-transition seen in our system, including figures S13-S17 referred here

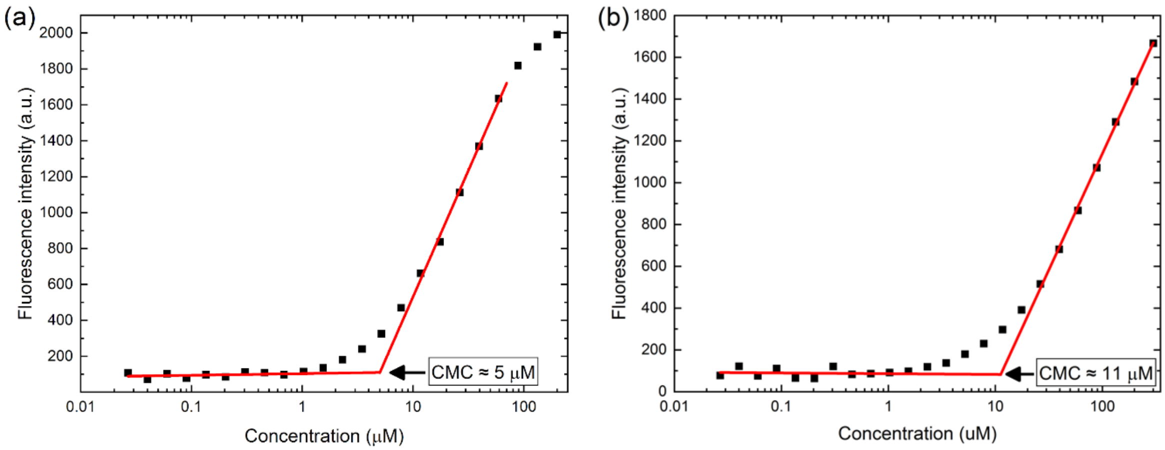

Figure S1. CMC measurements of (a) 2x12 and (b) 4x7 IDPAs. The CMC is taken to be the concentration at the intersection of the linear fits to the fluorescence intensity in both regimes. The concentrations are approximately 5 and 11 μM, respectively.

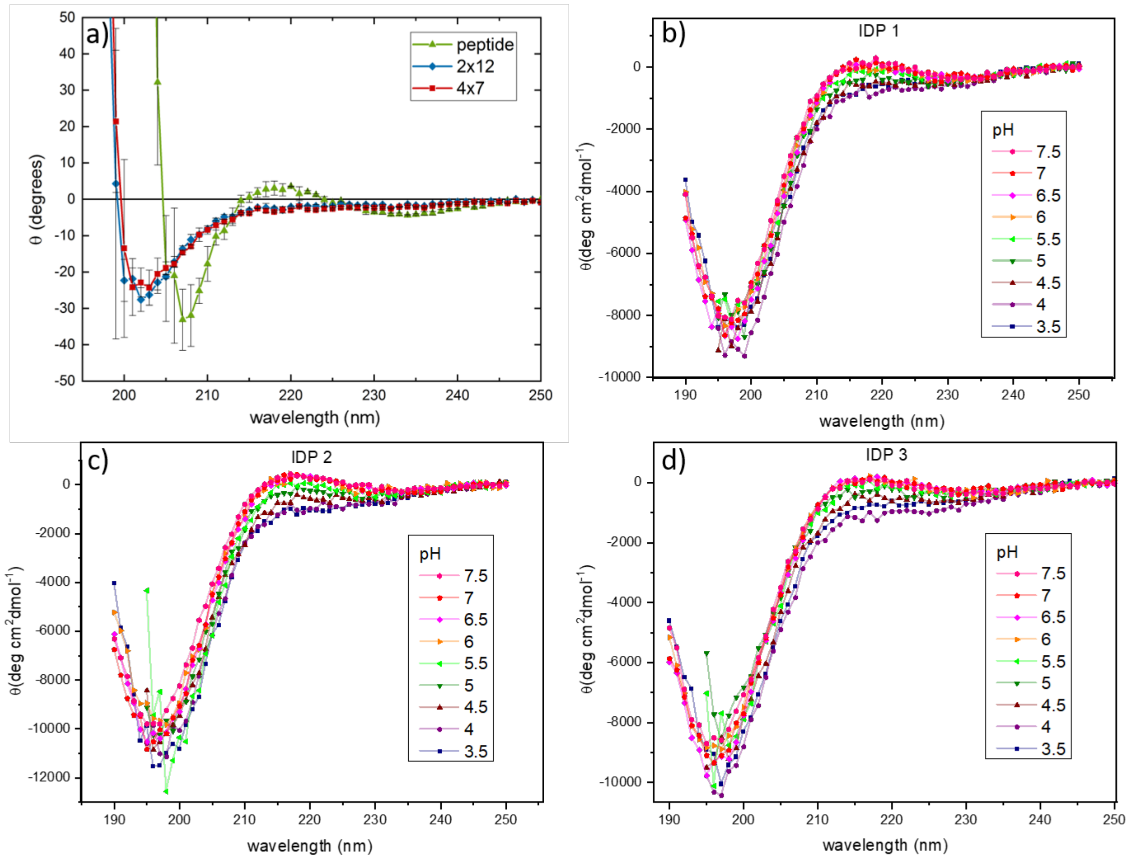

Figure S2. CD measurements a) of the IDPAs and the unconjugated peptide (IDP 1) in phosphate buffer (the buffer was changed due to strong absorption of the normal buffers used for sample preparation). b)-d) of the unconjugated peptide. Peptide was measured in the presence of 10mM sodium acetate, and sodium phosphate buffer for pH 3.5-5.5, pH 6-7.5, respectively. CD signal present random coil spectrum for all IDPs and IDPAs which indicates unstructured peptides/ peptide amphiphiles (disordered)[1] and no secondary structure for the relevant pHs.

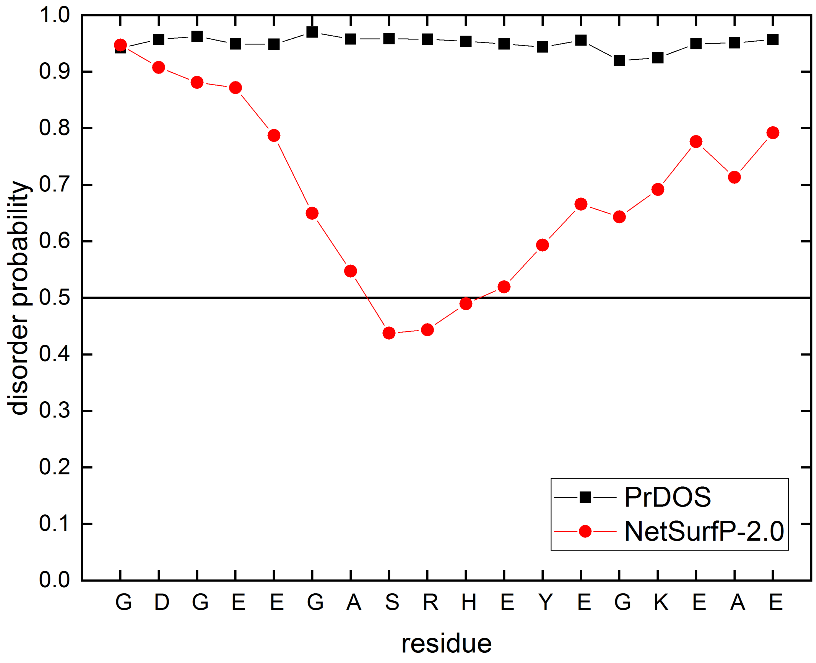

Figure S3. Results of software disorder predictions. The output of the PrDOS (black squares) and NetSurfP-2.0 (red circles), both showing a high probability for disorder of the peptide sequence.

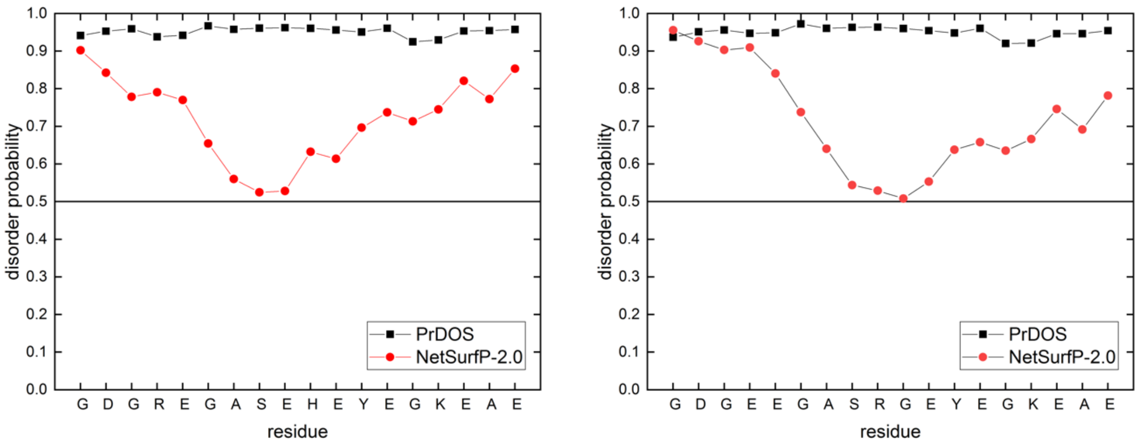

Figure S4. Results of software disorder predictions for the IDPA2 (left) and IDPA3 (right) sequences. The output of the PrDOS (black squares) and NetSurfP-2.0 (red circles), both showing a high probability for disorder of the peptide sequences.

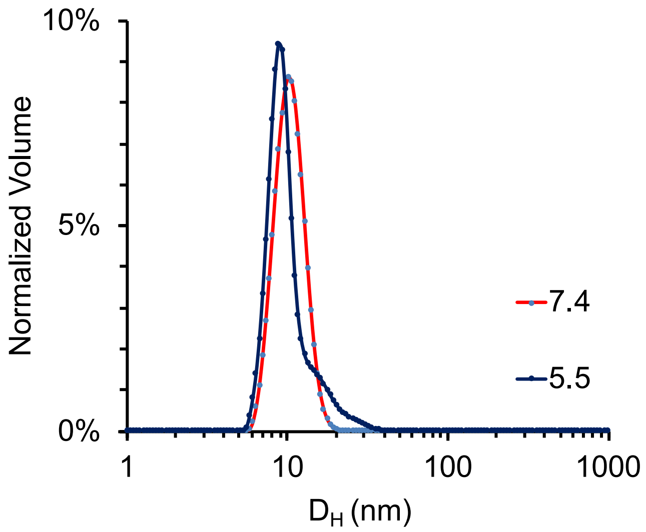

Figure S5. Dynamic light scattering measurements for the self-assembled PEG based amphiphiles (PEG-2x12) at pH 5.5 (blue line) and 7.4 (red line). Hydrodynamic diameter of about 10 nm is shown for both pH’s.

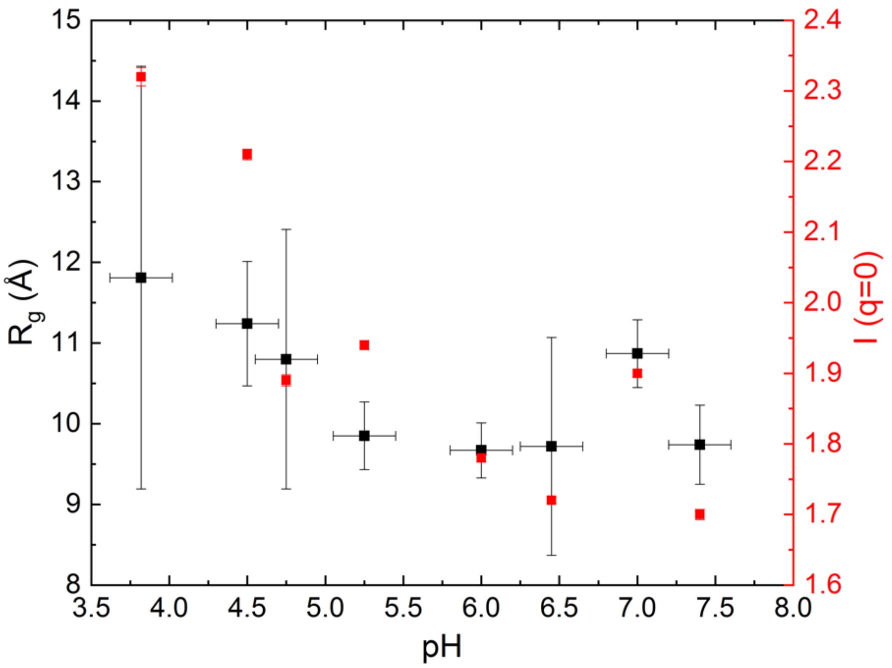

Figure S6. The radius of gyration extracted from the Guinier analysis at low *q* for the unconjugated IDP, as a function of pH. The observed slight increase in size is due to a decrease in repulsive interactions between the peptides closer to the pI, and an increase of forward scattering I_0.

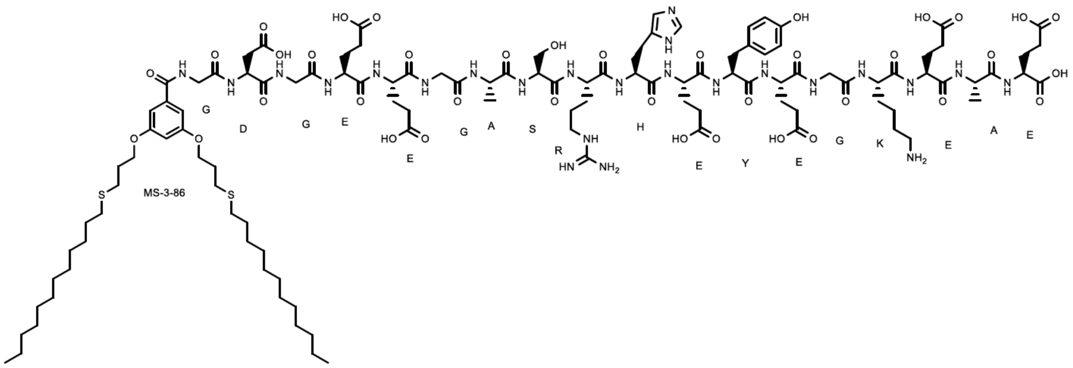

HPLC analysis:

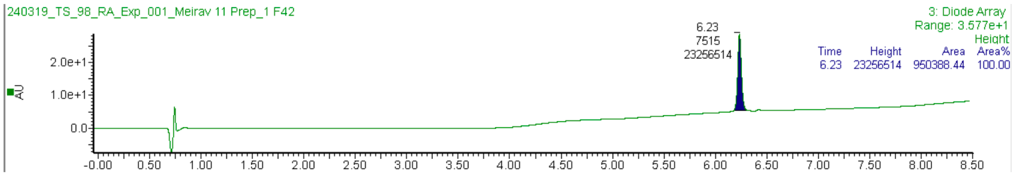

MS analysis:

Exact Mass: 2569.23

Detected Mass: ES mode +: 1286.78 [M+2H/2] and 858.17 [M+3H/3].

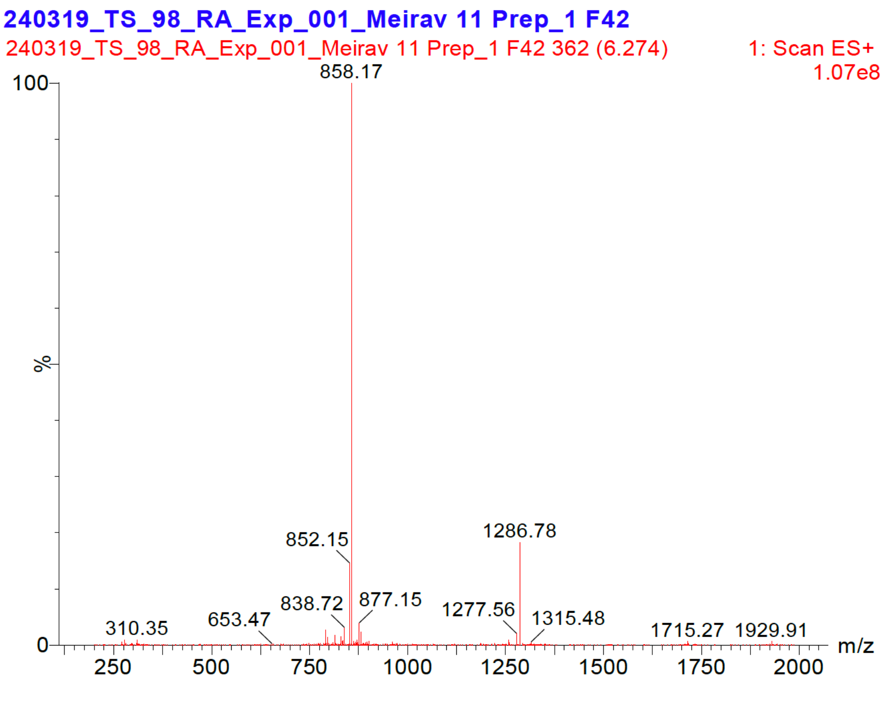

Figure S7. Chemical formula, HPLC Spectra and MS analysis for IDPA 1 2x12

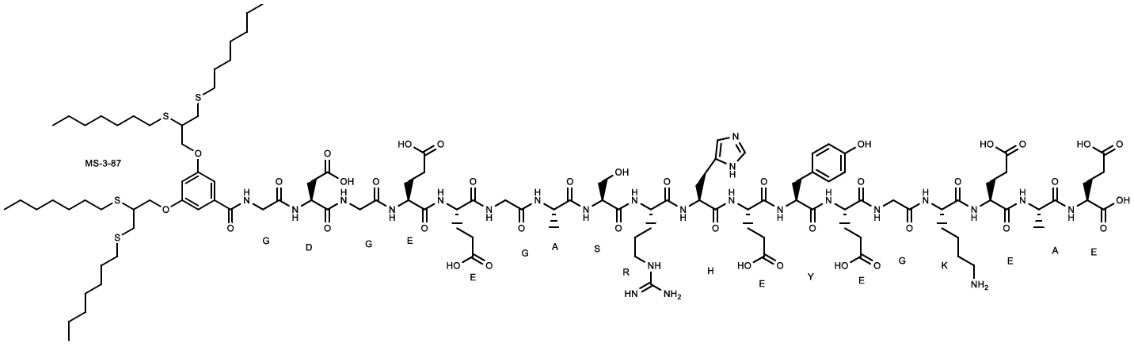

HPLC analysis:

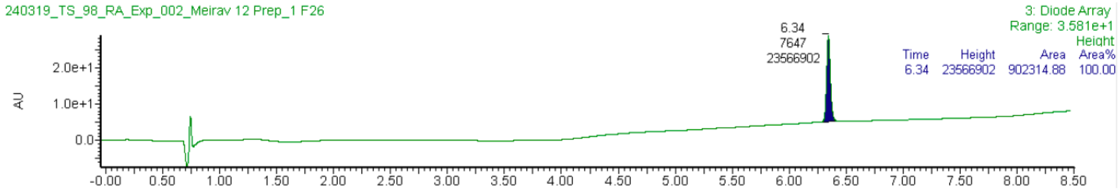

MS analysis:

Exact Mass: 2689.24

Detected Mass: ES mode +: 1346.78 [M+2H/2] and 898.22 [M+3H/3].

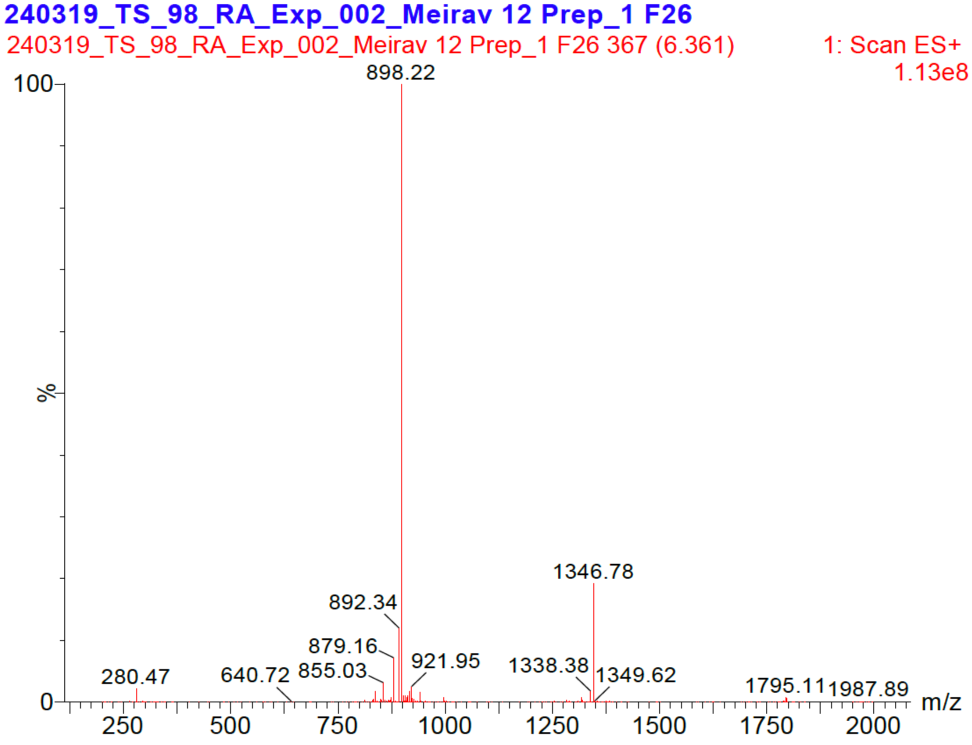

Figure S8. Chemical formula, HPLC Spectra and MS analysis for IDPA 1 4x7

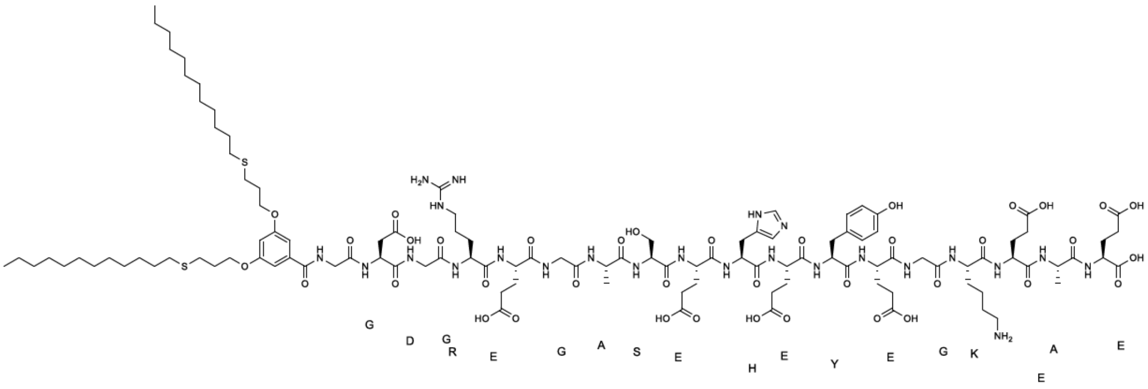

HPLC analysis:

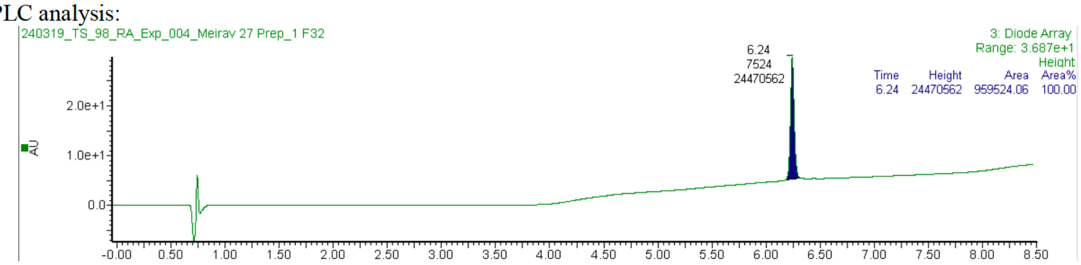

MS analysis:

Exact Mass: 2569.23

Detected Mass: ES mode +: 1286.78 [M+2H/2] and 858.17 [M+3H/3].

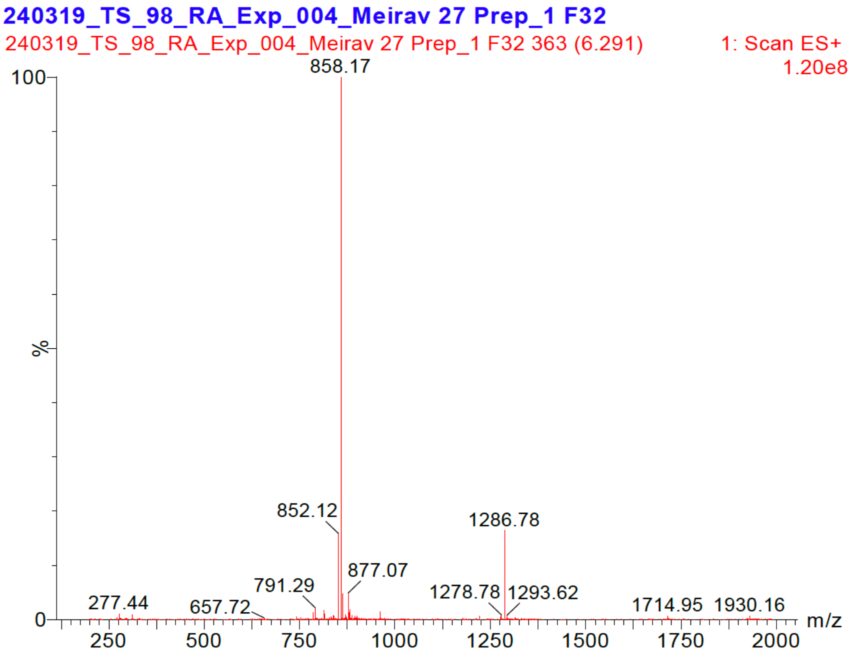

Figure S9. Chemical formula, HPLC Spectra and MS analysis IDPA 2 2x12

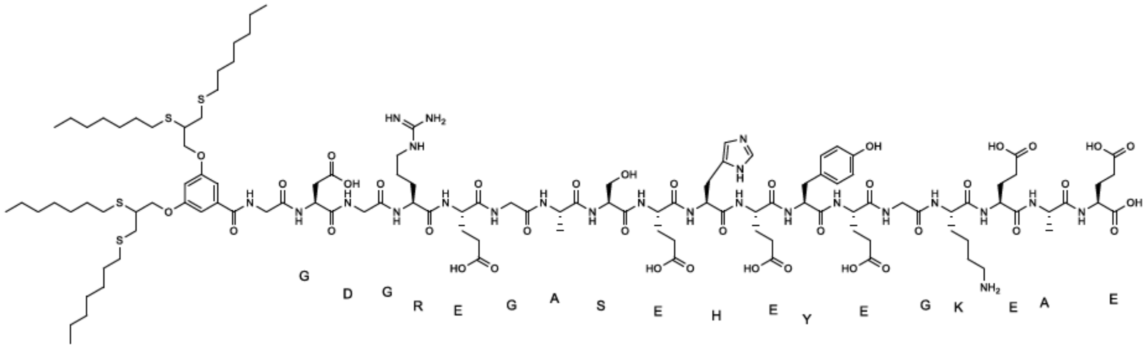

HPLC analysis:

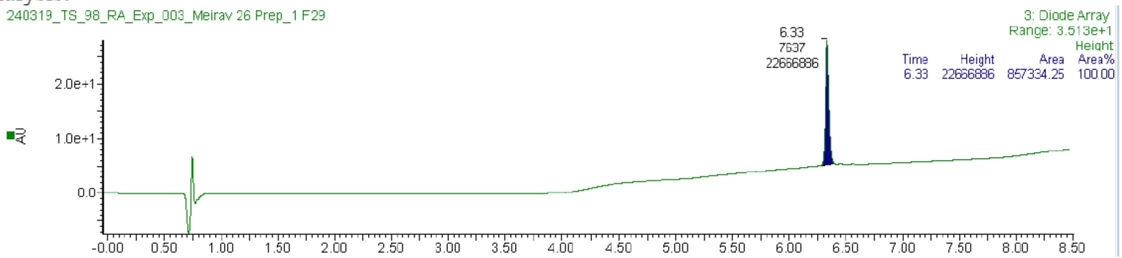

MS analysis:

Exact Mass: 2689.24

Detected Mass: ES mode +: 1346.78 [M+2H/2] and 898.22 [M+3H/3].

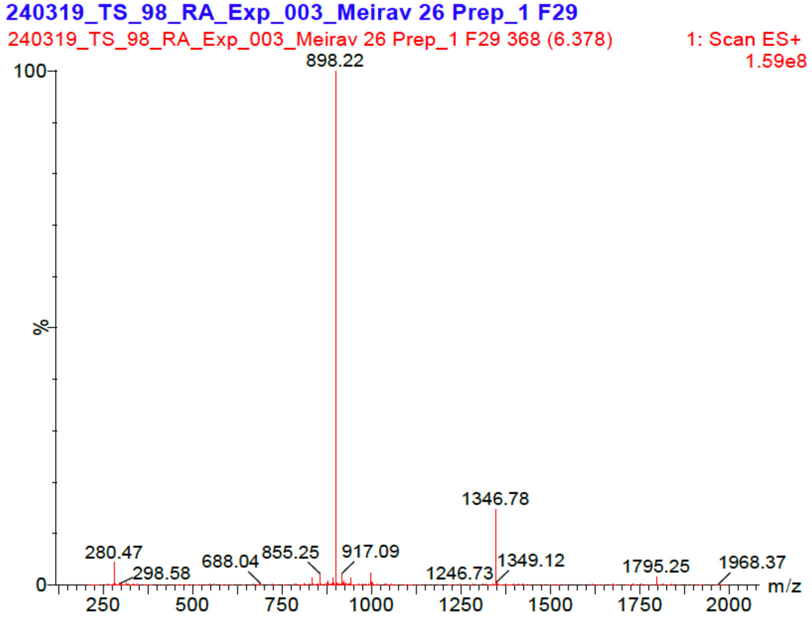

Figure S10. Chemical formula, HPLC Spectra and MS analysis IDPA 2 4x7

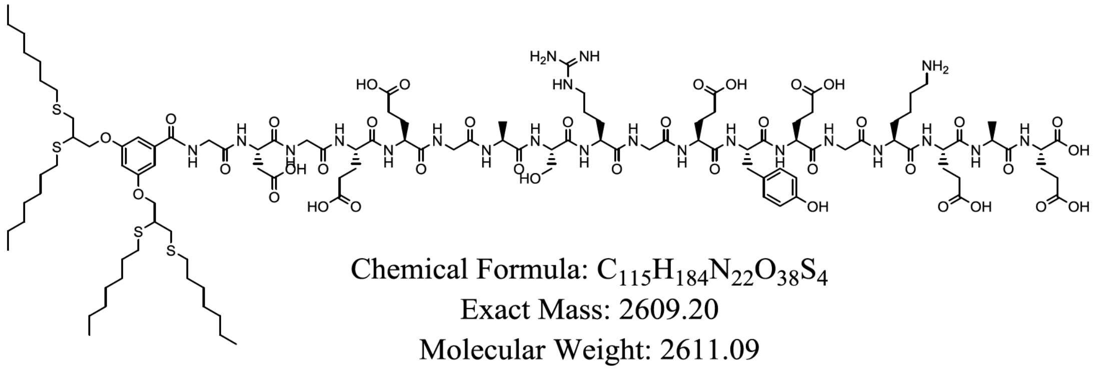

Chemical Formula: $C_{115}H_{184}N_{22}O_{38}S_4$
Exact Mass: 2609.20
Molecular Weight: 2611.09

HPLC analysis:

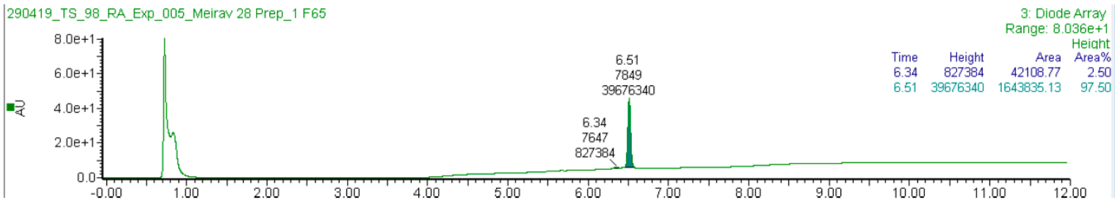

MS analysis:

Exact Mass: 2609.20
Detected Mass: ES mode +: 1306.83 [M+2H/2] and 871.47 [M+3H/3].

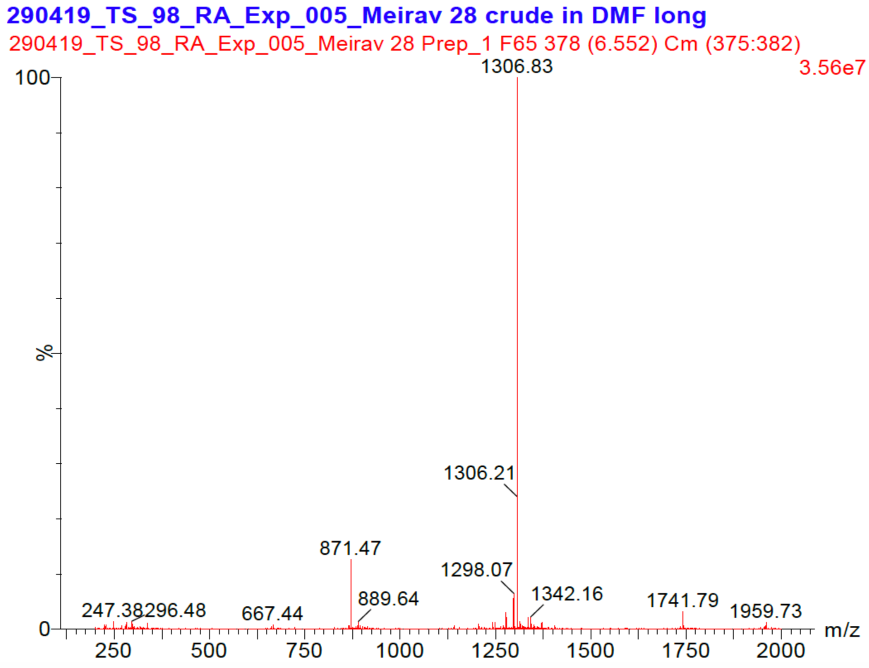

Figure S11. Chemical formula, HPLC Spectra and MS analysis for IDPA 3 4x7

(a)

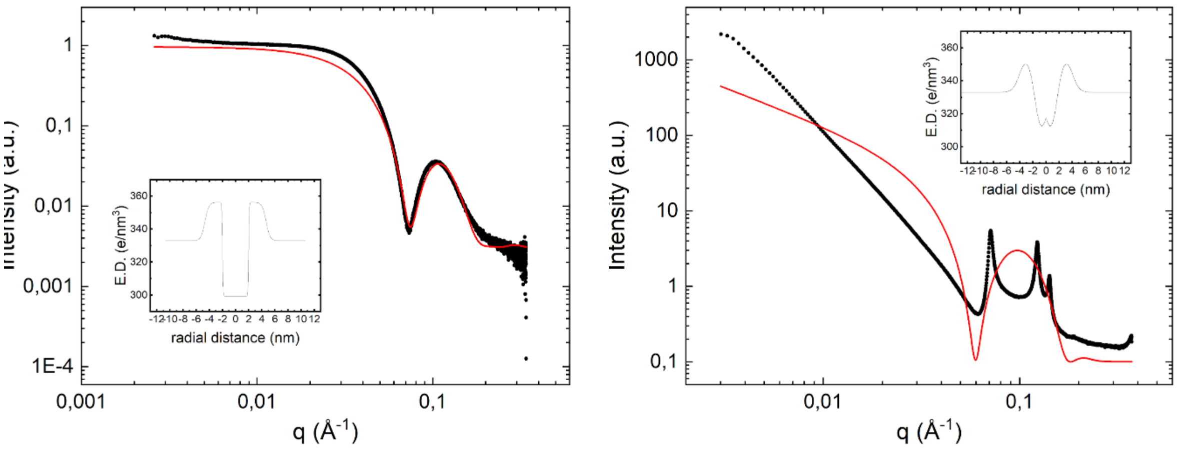

Figure S12. Core-Shell Form Factor Fits (a) Spherical Core-Shell Form Factor 2x12, pH 7.5 and (b) cylindrical Core-Shell Form Factor, 4x7, pH 3.

Theoretical arguments for the observed spherical-to-cylindrical micelle phase-transition seen in our system

Free-energy

We treat the transition between the two types of the self-assembled structures in terms of the free energy related to one molecule of the amphiphile, F. We assume the free-energy to be described by two competing contributions, the electrostatic free-energy, $F_E(Q)$, associated with the charge density of the peptides in headgroup region, and the bending energy, F_B, related to the deviations of the amphiphile monolayers constituting the micelles from their intrinsic curvature. The electrostatic energy depends on the peptide's charge, Q, which in turn depends on pH. The bending energy is charge independent and is set by the micellar shape only.

The transition between the spherical and cylindrical micelles is determined by the accompanied change of the system free energy, $\Delta F(Q)$, which can be presented as:

$$\Delta F(Q) = \Delta F_E(Q) + \Delta F_B \,, \qquad (1)$$

where $\Delta F_E(Q)$ and ΔF_B are the corresponding variations of the electrostatic and bending energy, respectively. To compute the free-energy contributions, we follow the procedure described in Ref. [2] and adapted to the system under consideration. Figure S7 shows a schematic representation of the cross-section of the spherical and cylindrical micelles assembled by the IDPAs. The figure also details the parameters used in the model, namely, the radius of hydrophobic core R, the width of the peptide shell δ, the charging length ξ (used for integration), the charge density ρ, Debye screening length in the shell and outside denoted by λ_s and λ_o respectively, the dielectric constants in the core, the shell, and the outside medium, denoted by ϵ_c, ϵ_s and ϵ_o respectively, and the electric potential ϕ, which has to be computed.

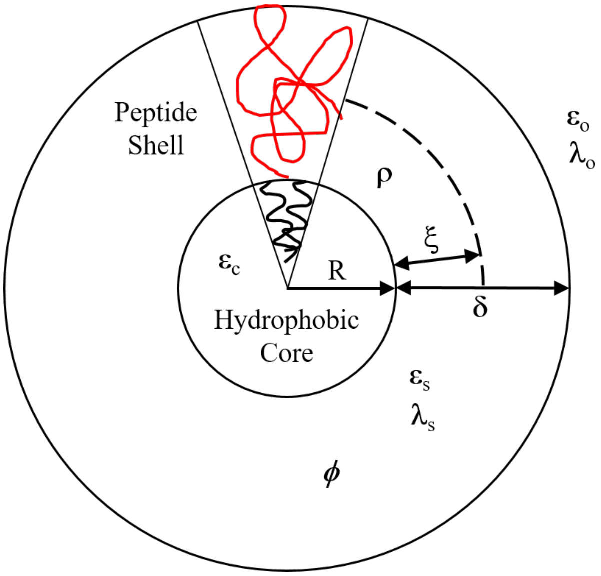

Figure S13. Schematic representation of a self-assembled spherical micelle of IDPAs.

To calculate the electrostatic free-energy of the micellar shell, we perform a procedure of a step-by-step charging of the space occupied by the shell. This procedure consists of sequential charging the concentric layers of infinitesimal thickness $d\xi$ lying one on top of the other. One step involves charging of a layer with radius, ξ, and area, $A(\xi)$, up to a charge $\rho(\xi)\, A(\xi)\, d\xi$, within the electric field produced by the previously charged layers characterized by the electric potential, $\phi(\xi)$. The electrostatic free-energy related to one amphiphilic molecule is given by:

$$F_E = \frac{A_m^s}{A_0} \int_0^\delta \phi(\xi)\, \rho(\xi)\, A(\xi)\, d\xi. \tag{2}$$

Here, A_0 is the reference plane area chosen within the amphiphilic monolayer forming the micelle and representing the micellar surface, A_m^s is the projection area of one amphiphile molecule on the reference plane. We take the reference surface to lie on the interface between the hydrophobic core and the hydrophilic shell of a micelle.

The electrostatic potential, $\phi(\xi)$, is found by solving the Poisson-Boltzmann equations in the three regions: the core, the shell, and outside the shell:

$$\nabla^2\phi(r) = \begin{cases} 0 & r < R \\ \lambda_s^{-2}\phi - \dfrac{\rho}{\epsilon_s} & R < r < R + \xi \\ \lambda_o^{-2}\phi & R + \xi < r \end{cases} \tag{3}$$

under the following boundary conditions:

$$\phi_c(0) = \text{const}\,; \qquad \phi_c(R) = \phi_s(R)\,; \qquad \epsilon_c \frac{\partial \phi_c}{\partial r}(R) = \epsilon_s \frac{\partial \phi_s}{\partial r}(R)$$
$$\phi_o(\infty) = 0\,; \qquad \phi_s(R+\xi) = \phi_o(R+\xi)\,; \quad \epsilon_s \frac{\partial \phi_s}{\partial r}(R+\xi) = \epsilon_o \frac{\partial \phi_o}{\partial r}(R+\xi) \tag{4}$$

To perform the calculation, the values of R, δ and the aggregation number must be known. We extract these values from the SAXS form factor fitting, as detailed in the main text. To the first approximation, the charge density can be calculated by equally distributing the total charge of the hydrophilic domain, Q, over the entire volume of the micellar shell. By computing the charge, Q, we consider its dependence on the pH of the surrounding solution, which is different for every specific IDPA.

We describe the bending energy per amphiphile molecule, F_B, by the relationship[3]:

$$F_B = \frac{1}{2} A_m^s \, \kappa \, (J - J_i)^2, \tag{5}$$

where κ is the bending modulus of the amphiphile monolayer, J is the mean curvature of the reference plane and J_i is the monolayer intrinsic curvature.

<u>Computing the free-energy</u>

Electrostatic free-energy

To compute the electrostatic free-energy, we first determine the electric potential, $\phi_s(\xi)$, underlying the charging of a layer of radius ξ by finding the solutions of Poisson-Boltzmann equations (Eq. 3) satisfying the boundary conditions (Eq. 4). We solve the equations, separately, for the spherical and cylindrical micelles, and the results depend on the system parameters ρ, R, λ_s, λ_o, ϵ_o, ϵ_s.

We perform the analysis under the following simplifying assumptions. The dielectric constants, the electrolyte concentrations and, consequently, the Debye screening lengths are taken equally for the headgroup shell region and the outside solution: $\epsilon_s = \epsilon_o$, $c_s = c_o$, $\lambda_s = \lambda_o$. Using the first assumption, and assuming the salt concentration in the solution outside is the same as inside the headgroup region, we set the radius of the reference plane, R, and the width of the peptide shell, δ, and the area per amphiphile molecule, A_m^s, are equal for the spherical and cylindrical micelles. The charge density, ρ, is uniform over the micellar shell's width and, hence, does not depend on ξ.

Under these assumptions, obtain for the area per molecule, $A_m^s = \frac{4\pi R^2}{N_m}$, where N_m is the number of molecules (i.e., aggregation number). The volume per molecule, V_m^s, is given for a spherical micelle by, $V_m^s = A_m^s \delta \left(1 + \frac{\delta}{R} + \frac{\delta^2}{3R^2}\right)$, and for a cylindrical micelle by, $V_m^c = A_m^s \delta \left(1 + \frac{\delta}{2R}\right)$.

The corresponding charge densities in the spherical and cylindrical micelles are given, respectively, by $\rho_s = \frac{Q}{V_m^s}$ and $\rho_c = \frac{Q}{V_m^c}$.

Using the computed potential and charge density, we calculate the electrostatic free-energy using Eq. (2) for a specified salt concentration in solution.

Elastic free-energy

Computing the contribution to the total free-energy of bending (Eq. 5) is straightforward for a given intrinsic curvature, J_i, and bending modulus, κ, of the amphiphile monolayer. However, since we have no experimentally determined values of these parameters for our system, we will treat the difference in elastic free-energy between the spherical and cylindrical micelles $\Delta F_B = \frac{1}{2}\frac{\kappa a}{R}\left(\frac{3}{R} - 2J_i\right)$, as a single parameter and find phase boundaries for different values of ΔF_B (lines of $\Delta F = 0$). The value of this parameter is determined by using a combination of physically relevant values for the intrinsic curvature and bending rigidity. For the model to predict a phase-transition, the intrinsic curvature, J_i, must be smaller than $\frac{3}{2R}$ (the midpoint between the curvature of a cylinder and a sphere). A reasonable value for the bending modulus will be taken between 10 and 100 k_BT, based on values obtained experimentally for monolayers of the previously studied amphiphiles[3].

Non-specific peptide-peptide interactions

From the data presented in the main text, it is apparent that alternative sequences self-assembled differently, even when the net charge is kept constant. The model described in the previous section does not take into account a possible interaction between the amphiphile molecules related to the sequence specificity. Therefore, we expand the model by explicitly including the energy of this interaction into the energy balance of the micellar transition,

$$\Delta F(Q) = \Delta F_E(Q) + \Delta F_B + \Delta F_{SS}\,, \qquad (6)$$

where ΔF_{SS} stands for the sequence-specific interaction between neighboring polyampholytes due to the distribution of charges on the peptide. Relative conformations of neighboring peptides can result in an attractive interaction, as was previously demonstrated for other intrinsically disordered proteins [4–8]

We qualitatively model this using the "Handshake analysis", previously described in Refs. [4,5]. This analysis generates a heat-map of electrostatic interaction between different segments of interacting peptides, revealing "attractive conformations". The calculation of electrostatically interacting segments is performed by setting the peptide persistence length (PL), which determines the segment size (w amino acids are in

contact in each segment), and the number of nearest neighbors (NN) that interact within that segment (each amino acid interacts with $2m + 1$ amino acids from the opposite segment)

$$\Delta E(n_1, n_2) = k_e \sum_{i=-w/2}^{w/2} \sum_{j=-m}^{m} \frac{eZ_1(n_1 + i)eZ_2(n_2 + i - j)}{|r_1(n_1 + i) - r_2(n_2 + i - j)|}. \quad (7)$$

The outcome of the analysis is a prediction regarding the possibility of a preferred conformation of attractively interacting peptides, as well as an approximation of the strength of the interaction. Figure S8-S9 show the analysis results for two different sets of PL and NN.

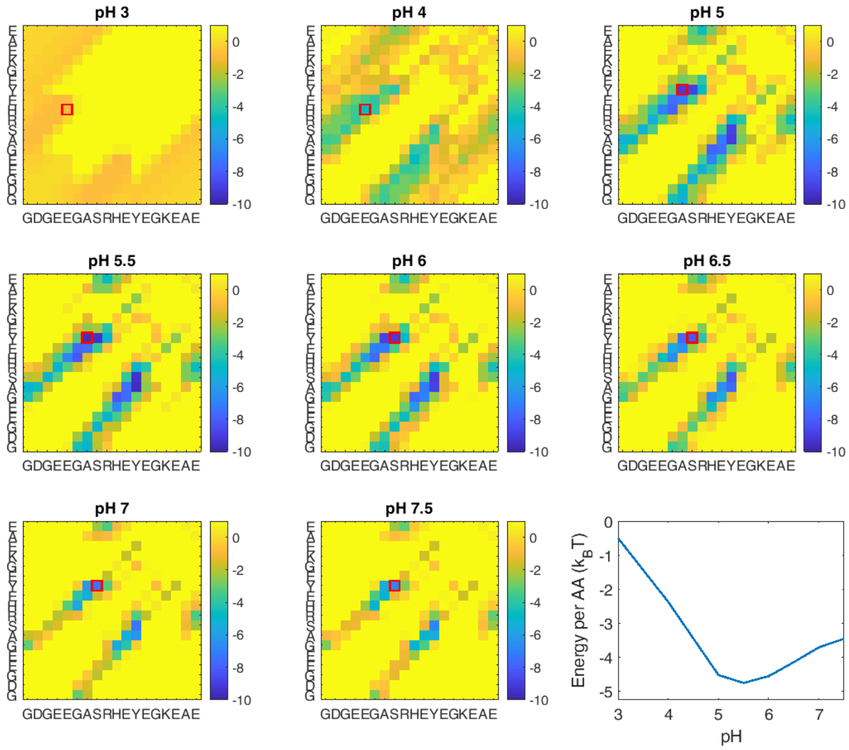

Figure S14. Handshake analysis heat-maps, for PL = 3 and NN = 3, showing the preferred attractive sliding-conformations as a function of pH. The color bar indicates the interaction strength in k_BT_{300K}. The bottom right graph shows our approximation of the strength of interaction at each pH, chosen to be the strongest point of attractive interaction (marked in red on the heat-maps).

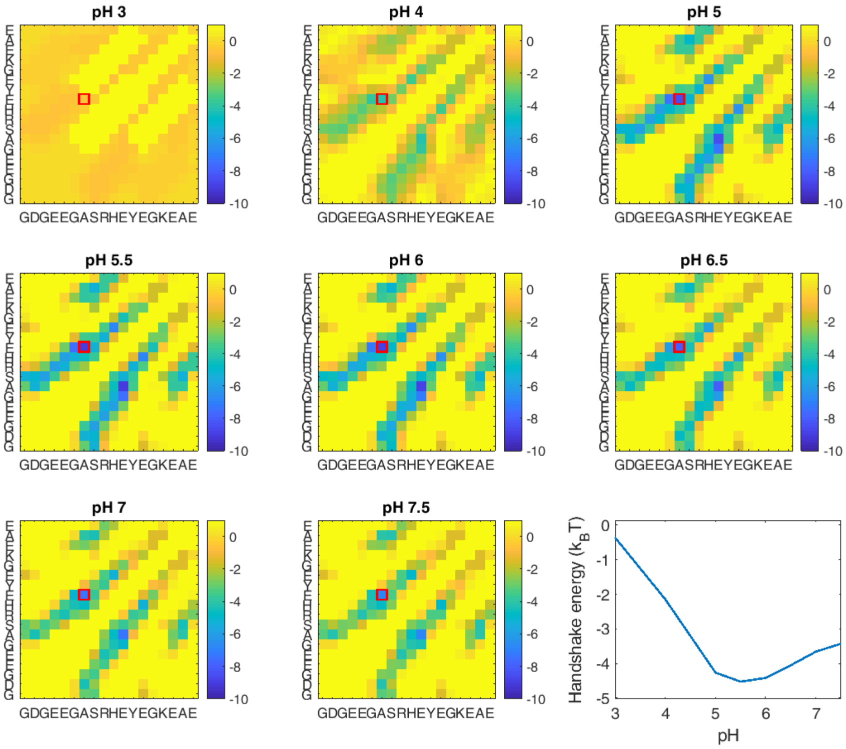

Figure S15. Handshake analysis heat-maps, for PL = 2 and NN = 1, showing the preferred attractive sliding-conformations as a function of pH. The color bar indicates the interaction strength in $k_B T_{300K}$. The bottom right graph shows our approximation of the strength of interaction at each pH, chosen to be the strongest point of attractive interaction (marked in red on the heat-maps).

The diagonal lines that increase in strength from pH 10 to 5.5 (dark color diagonal line) are a specific shifted conformation of adjacent peptides that is favorable. In this case, a shift of about five amino acids produces attractive interaction between parallel peptides, and the strength of the interaction is approximated by the strongest contact point (darkest square on the diagonal). Using these values, we can approximate the dependence of the handshake interaction on pH (bottom right panel of Figure S8-S9). Mapping this dependence is useful for assessing the contribution of the conformation-specific interaction to the overall interaction leading to the phase-transition, at least semi-quantitatively.

Sequence variants

When considering the sensitivity of the phase to pH, and more specifically, the phase-transition being located somewhere between pH 4 and 6, a possible explanation can be the histidine residue's charging state. Histidine is the only amino acid with a side chain group that gets protonated in this pH range.

We used the handshake analysis tool to design slightly modified headgroup sequences and predict their behavior of interaction. Two variants of the original sequence were produced: IDPA2 – swapping the

Glutamic Acid at position 4 with the Arginine at position 9 and IDPA3 – replacing the Histidine at position 10 with a Glycine residue. It is important to note that the total (and net) charge of IDPA3 has changed, as it will no longer have an additional positive charge below pH 6. IDPA2, on the other hand, retains the same total (and net) charge, but has a different distribution of charges along the sequence.

The handshake analysis also predicts a weaker interaction for the same conformation chosen for the original sequence (Figure). The IDPA2 variation was predicted by the analysis to abolish the favorable interaction of the conformation seen in the original (Figure).

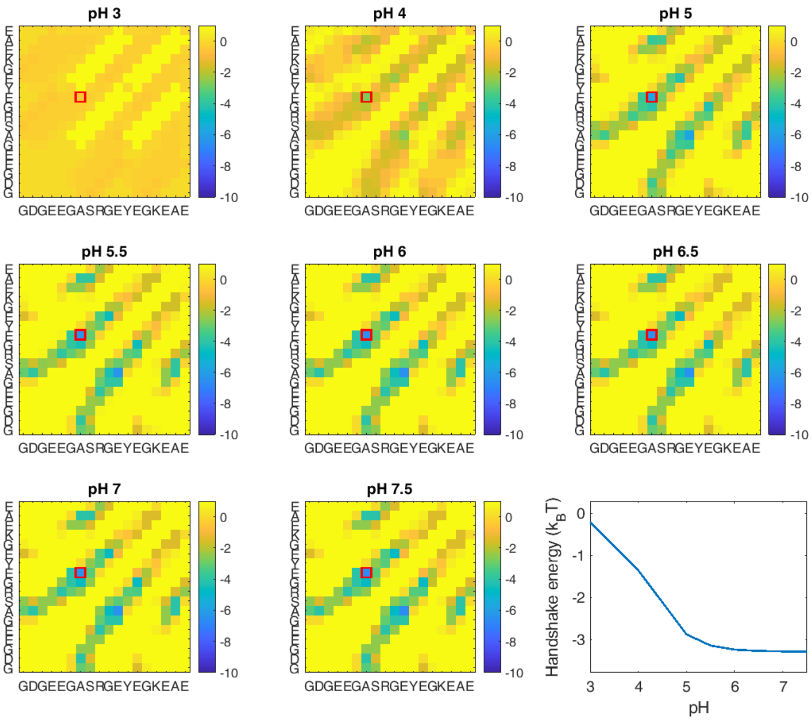

Figure S16 The handshake analysis, with PL = 2 and NN = 1, for the IDPA3 sequence. The interaction energy is weaker than for the original sequence, and there is no longer a minimum around pH 5.5. The color bar indicates the interaction strength in $k_B T_{300K}$. The bottom right graph shows our approximation of the strength of interaction at each pH, chosen to be the strongest point of attractive interaction (marked in red on the heat-maps).

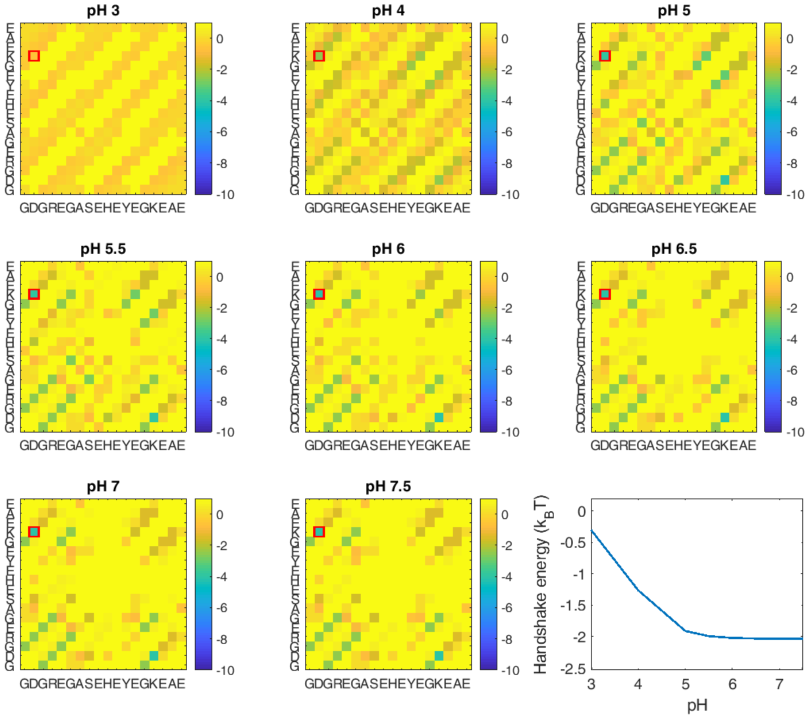

Figure S17. The handshake analysis, with PL = 2 and NN = 1, for the IDPA2 sequence. The switching has abolished the attractive conformation from the original sequence. The color bar indicates the interaction strength in $k_B T_{300K}$. The bottom right graph shows our approximation of the strength of interaction at each pH, chosen to be the strongest point of attractive interaction (marked in red on the heat-maps).

Both of these variants are still considered highly disordered and are assigned the random coil conformation by the bioinformatic predictors (figure S4).

References:

1. Kelly, S. M., Jess, T. J. & Price, N. C. How to study proteins by circular dichroism. *Biochim. Biophys. Acta - Proteins Proteomics* **1751**, 119–139 (2005).

2. Lerche, D., Kozlov, M. M. & Markin, V. S. Electrostatic free energy and spontaneous curvature of spherical charged layered membrane. *Biorheology* **24**, 23–34 (1987).

3. Bassereau, P. & Sens, P. *Physics of biological membranes*. *Physics of Biological Membranes* (Springer International Publishing, 2018). doi:10.1007/978-3-030-00630-3.

4. Beck, R., Deek, J., Jones, J. B. & Safinya, C. R. Gel-expanded to gel-condensed transition in neurofilament networks revealed by direct force measurements. *Nat. Mater.* **9**, 40–46 (2010).

5. Kornreich, M., Malka-Gibor, E., Laser-Azogui, A., Doron, O., Herrmann, H. & Beck, R. Composite bottlebrush mechanics: α-internexin fine-tunes neurofilament network properties. *Soft Matter* **11**, 5839–5849 (2015).

6. Kornreich, M., Malka-Gibor, E., Zuker, B., Laser-Azogui, A. & Beck, R. Neurofilaments Function as Shock Absorbers: Compression Response Arising from Disordered Proteins. *Phys. Rev. Lett.* **117**, 148101 (2016).

7. Kornreich, M., Avinery, R., Malka-Gibor, E., Laser-Azogui, A. & Beck, R. Order and disorder in intermediate filament proteins. *FEBS Lett.* **589**, 2464–76 (2015).

8. Malka-Gibor, E., Kornreich, M., Laser-Azogui, A., Doron, O., Zingerman-Koladko, I., Harapin, J., Medalia, O. & Beck, R. Phosphorylation-Induced Mechanical Regulation of Intrinsically Disordered Neurofilament Proteins. *Biophys. J.* **112**, 892–900 (2017).
